# Assessing an effective undergraduate module teaching applied bioinformatics to biology students

**DOI:** 10.1371/journal.pcbi.1005872

**Published:** 2018-01-11

**Authors:** Andreas Madlung

**Affiliations:** University of Puget Sound, Department of Biology, Tacoma, Washington; Genome Quebec, CANADA

## Abstract

Applied bioinformatics skills are becoming ever more indispensable for biologists, yet incorporation of these skills into the undergraduate biology curriculum is lagging behind, in part due to a lack of instructors willing and able to teach basic bioinformatics in classes that don’t specifically focus on quantitative skill development, such as statistics or computer sciences. To help undergraduate course instructors who themselves did not learn bioinformatics as part of their own education and are hesitant to plunge into teaching big data analysis, a module was developed that is written in plain-enough language, using publicly available computing tools and data, to allow novice instructors to teach next-generation sequence analysis to upper-level undergraduate students. To determine if the module allowed students to develop a better understanding of and appreciation for applied bioinformatics, various tools were developed and employed to assess the impact of the module. This article describes both the module and its assessment. Students found the activity valuable for their education and, in focus group discussions, emphasized that they saw a need for more and earlier instruction of big data analysis as part of the undergraduate biology curriculum.

## Introduction

In the post-genomic era, bioinformatic analysis skills have become an all but unavoidable need for life scientists [1] who are tasked with learning and often teaching skills and tools that they themselves may not have acquired during their own training [2]. In a 2017 survey of more than 700 biologists funded by the American National Science Foundation, more than 90% of respondents stated that they were, or soon would be, working with large data sets that required high-performance computing [3]. These same researchers listed training in data analysis tools and bioinformatics as the most urgent and unmet need they had to address to successfully complete their research projects [3].

Bioinformatics is a complex field of study. Life scientists rely on bioinformaticists to help them analyze big data sets that require knowledge of how to format and parse through data files and how to write computer scripts and programs that can connect existing software applications. Bioinformaticists have to apply software tools that don’t have graphical user interfaces, navigate the use of high-power computer clusters, and often have to have at least basic system administration knowledge. In addition, bioinformaticists help with experimental design, statistical analysis, and data visualization. However, according to many practitioners in the field and textbooks on bioinformatics, bioinformaticists are first and foremost computer scientists who use computer programming tools to develop new algorithms and programs useful for biological applications and are not primarily data analysts serving the needs of biologists [4–7]. In college teaching, this dichotomy is often reflected in the choice of which department, if any, might offer a bioinformatics course, what content such a course covers, and what kind of prerequisite knowledge is expected of students taking the course [6,8]. Some authors have tried to clarify the different aspects of bioinformatics by subdividing the field of bioinformatics depending on interest, training, and focus into “bioinformatics users,” “bioinformatics scientists,” and “bioinformatics engineers” [9]. For all practical purposes, in life science research, the ubiquity of big data and the need for biocomputing skills means that biologists either have to learn the use of bioinformatics tools themselves or hire (or collaborate with) a specialist to help them with data analysis. Biologists at all levels are well served if they can analyze their data on their own, or at least speak the same technical language as their collaborators who help them with data analysis. As of 2015, fewer than 30 institutions throughout the United States were reported to offer bioinformatics degree-granting programs at the undergraduate level [9]; however, if including the term “computational biology” into a search, 72 such programs were reported [10]. How much training in bioinformatics standard biology programs require was not reported in these studies.

It is before this backdrop that biologists have started developing workshops, online tutorials, and course materials [11] to familiarize mostly current graduate students and postdocs with the applied kind of bioinformatics that, for example, allows the analysis of next-generation sequencing data to address problems in molecular biology, physiology, population genetics, or evolutionary biology. However, in order to be able to use next-generation sequence analysis tools or even just to be able to understand the language of many tutorials and the syntax of simple computer code, novices must first learn some basic computer science, the use of Unix, shell scripting, and the use of the Linux computer operating system. Only then can they start using tools for DNA or RNA sequence analysis, including genome assembly, RNAseq (transcriptome) analysis, or SNP detection for trait mapping and the analysis of evolutionary questions.

Because bioinformatics is not traditionally a subject that most undergraduate biology students learn within the core classes of their biology curriculum and because students often have preconceived notions that biology is, from among the sciences, the subject that least relies on large-scale mathematical skills, it is important to monitor students’ attitudes towards the introduction of computational biology into traditionally less-computational biology courses, since such preconceived notions can affect student learning [12]. It has been shown that one way to assess student learning is to measure how their attitude towards a particular field of study changes over time as they are being exposed to this field [13]. Such attitude change can be measured on a continuum between novice and expert by comparing student responses to a specific type of question—before and after they engage in an activity—with the answers that an expert would give. Participation in a course, workshop, or activity should ideally lead to changes in students’ attitude along this continuum towards that of an expert.

The Colorado Learning Attitudes about Science Survey (CLASS; http://www.colorado.edu/sei/class/) was based on studying novice-to-expert development and specifically designed to measure student attitude development in three areas of learning [13]. The first area measures to what degree a respondent views the field at hand as a collection of unrelated facts as opposed to a cohesive set of concepts. The second distinguishes between the novice-like perception that facts in the discipline of study are fixed versus an expert-like view that sees facts as results of experimentation and analysis. The third area assesses to what degree problem-solving abilities are developed along the novice-to-expert line [14]. CLASS surveys have been successfully used to specifically determine how students’ perceptions impact their learning, as well as to assess pedagogical tools that are designed to help students mature in their understanding and appreciation of the sciences [14]. CLASS surveys have previously been developed to assess classes in physics [15], chemistry [16], biology [14], and computer science [17].

This paper describes a teaching module that was developed for educators of upper-level undergraduate biology majors, who—like their students—have little or no prior knowledge of computer science, programming, or working on the command line. The module was designed to achieve the following three learning objectives: (a) teach students basic command-line computing and bioinformatics skills, (b) motivate students to develop an interest in bioinformatics approaches to address biological questions, and (c) allow students to develop an appreciation for the use of bioinformatics in modern biology. The module developed for this study was therefore written in a way that allows teaching faculty with no experience in next-generation sequence analysis to prerun the module and to teach themselves enough to be able to use the module in class. The module was piloted in the fall of 2014, refined after its first use, and subsequently assessed in three separate upper-level elective courses (twice in a molecular biology course and once in a plant physiology course) taught by the author. It was also tested by two additional novice educators and taught at a second institution in an upper-level biology class. The assessment of the module was based on the following four aspects: the use of a pre- and postmodule questionnaire for students, the comparison of student answers to a group of “experts,” a formal focus group, and student transcript analysis. The questionnaire used for the assessment was modeled after other validated CLASS assessment tools [14–17]. The results of our assessment suggest that the module increases student learning, increases student interest in applied bioinformatics, and is equally effective for students at all levels of preparation as measured by their overall college performance.

## Methods

### Ethics statement

This study was approved for exempt status by the Institutional Review Board of the University of Puget Sound (approval # 0708–018). Participants were also asked to provide written permission for the use of their data and for the use of their college transcript by the investigator.

### Approach

The goals of this study were three-fold, as follows: first, to design a teaching module that requires no previous computer science skills and introduces students (and potentially their teachers) to applied bioinformatics methods; second, to rigorously assess the value of this module to student learning; and third, to gain insights from the assessment that can guide future curricular decisions for undergraduate biology education.

### Institutional details and cohort selection

The study was performed at the University of Puget Sound, a small, selective liberal arts college in Washington State (US). Puget Sound graduates about 60 to 70 majors in biology per year. To balance the need for a large-enough sample size and a fast-enough study time, three upper-level classes all taught by the author in successive semesters from spring 2015 (N = 24), fall 2015 (N = 15), and spring 2016 (N = 12) were chosen as the student cohort. Experts, whose survey answers are used as a control group in CLASS studies, were chosen to fit the following description: these experts were natural scientists by training, had a PhD degree or were within a year of attaining their PhD, were interested in college teaching and had at least some teaching experience, and could be considered primarily as life scientists (as opposed to bioinformaticists, computer scientists, or strictly computational biologists). Thus, those providing the expert opinion were not experts in bioinformatics but experts in a field of life sciences that required or might require the use of bioinformatics tools. This group was selected to enrich the expert group in scientists who didn’t choose to become professional bioinformaticists out of interest in the field of bioinformatics but instead supposedly resembled the participating students in their academic career paths. The total number of experts participating was 50.

### Module design

The module created for this study demonstrates to students the use of next-generation sequencing of RNA—a technique known as RNAseq. This method allows the analysis of the entirety of all active genes in an organism (the transcriptome), for example in response to a stimulus or during growth and development. While plenty of online tutorials exist explaining the use of the software employed here (the so-called “Tuxedo pipeline”), most tutorials are not written in a way that is accessible to complete novices, as in the example of the leading method publication in the journal *Nature Protocols* describing the Tuxedo pipeline [18].

**Fig 1:**
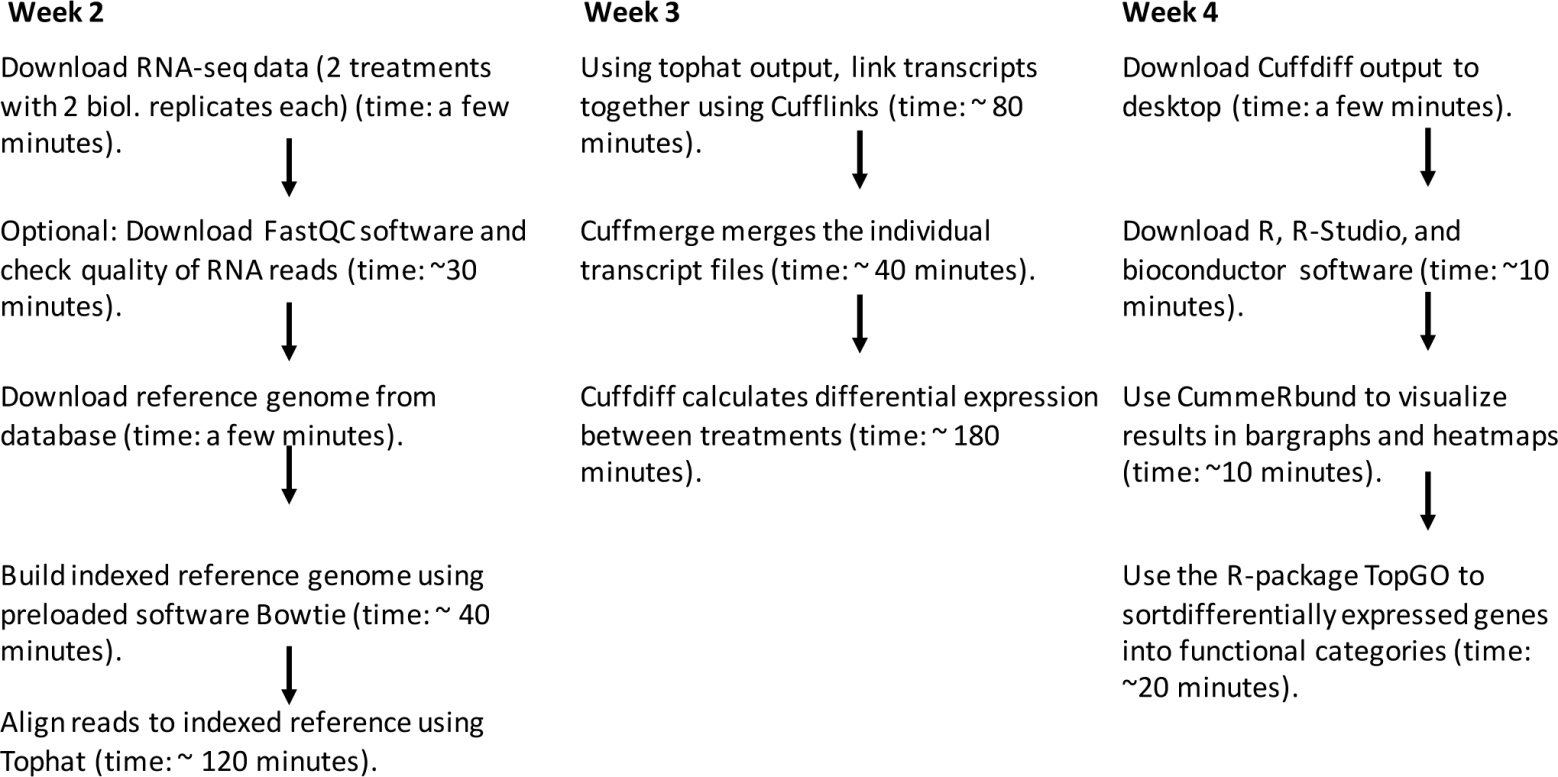
Flowchart depicting the compute pipeline used in the module. In Week 1, all introductory Unix exercises are performed on the student’s computer without the need for cloud computing or a Linux cluster. In Week 1, students also sign up for a free CyVerse account and start up a virtual machine. In Weeks 2 and 3, most of the cloud computing steps are performed. The approximate compute times for each step are listed based on the use of a virtual machine with one CPU (“small” CyVerse instance). In between compute steps, students write scripts, format and verify data using command-line Unix tools, read the rationale behind each step, and spend some time watching a YouTube video (for the FastQC tutorial) and getting familiar with specific help forums for bioinformatics questions, such as Biostars. Depending on student preparedness and engagement, each lab session can be completed in 2.5 to 4 hours. Using more CPUs can speed up the compute time significantly for Tophat and CuffDiff analyses, which are in the current lab set-up “overnight” steps separating labs. CPU, central processing unit.

**Fig 2:**
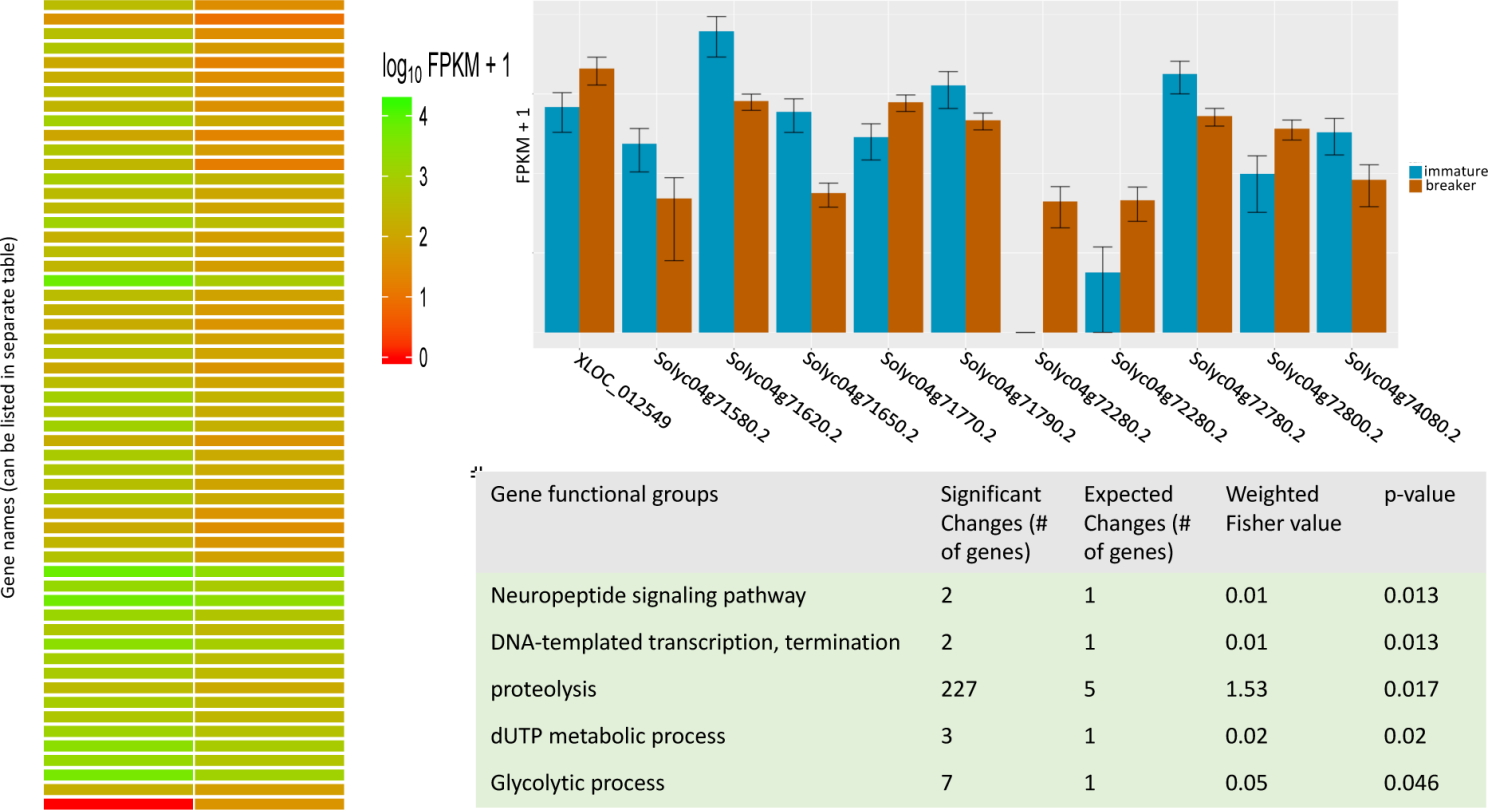
Sample figures and tables from RNAseq analysis in the described module. The RNAseq pipeline creates a variety of figures that students can customize by changing the R-script that creates them. CummeRbund analysis creates the heatmap (left) and the bar chart (top right). Using the R-package TopGO, students calculate the probabilities (using Fisher exact tests) that certain functional categories are enriched in differentially expressed genes from the RNAseq analysis (table bottom right). Abbreviations: dUTP, 2´-Deoxyuridine, 5´-Triphosphate; FPKM, Fragments Per Kilobase of transcript per Million mapped reads.

The experiments conducted in this module begin by downloading RNA sequence data from a repository. The data in this experiment compare transcriptomes of unripe versus ripe tomatoes. Comparison between data sets should therefore allow the identification of genes that are important in the ripening process. After data are downloaded, the RNA sequences (the so-called “reads”) are quality checked and computationally aligned to the tomato reference genome. The number of reads for each gene is compared between the two developmental stages (unripe vs ripe) and assessed for statistical significance. Fig 1 describes this process along with the logistics of the module in flowchart format. Eventually, genes that are differentially expressed between the two samples are categorized by function and graphically displayed. Fig 2 shows examples of student analyses of the data set used in the module.

The format of the module was designed to fit 4 four-hour–long lab sessions and written in the style of a self-guided tutorial. Each of the four parts is self-contained so that students have minimal homework to do between sessions. In this study, students worked on their own during regularly scheduled lab sessions with the instructor (author of the module) present. Depending on the computational power of the instance (i.e., a virtual computer) chosen, large-scale calculation steps take between a few minutes and several hours. Larger instances with more memory can reduce the compute time of the most time-consuming step to 20 minutes. If the module is used as designed for 4 four-hour–long labs, the longest compute times fall at the end of a lab session so that they can be run in the cloud after the student has left the lab. Fig 1 shows estimated compute times per step. As an alternative to multiple lab sessions, students can elect to do the entire tutorial on their own time and on their own computer, or the project can be completed in a workshop format. All required software and hardware (except for a regular desktop or laptop computer) is freely available. The raw data can be downloaded from a publicly accessible file transfer protocol (FTP) site. The high-performance computing platform used here is the freely accessible CyVerse (formerly iPlant) cloud computing cluster, and the required software is preloaded on a so-called image (essentially a virtual external hard drive), to which students connect once they have registered for their CyVerse account.

During the first session, students sign up for and activate a private account, perform a tutorial in which they learn basic commands in the command-line scripting language Unix, which is preinstalled on Macintosh computers and is also preloaded on CyVerse. In the second and third session, students are introduced to the main project, in which they perform the comparison of the transcriptome of unripe versus ripe tomato fruit. This analysis requires them to use a variety of software programs and file manipulation Unix commands. During the fourth session, students download and install the free statistics and graphing software R, which they then use to manipulate the output from the previous session, create bar graphs and heatmaps of their gene expression data, and finally perform so-called gene ontology analysis. Using R, they categorize the genes differentially expressed between unripe and ripe tomatoes into functional classes and calculate the statistical significance, with which these functional groups are overrepresented among all functions within the differentially expressed genes. The module contains various pre- and postlabs and a final assignment, but the material in the assessment of the module did not appear on any class exams. The cumulative value of these assignments in the context of the whole session in which the module was taught made up about 5% of the students’ overall grade for the course. The complete teaching material is available in S1 Supporting information.

### Instrument design

The questionnaire was modeled closely after the validated CLASS assessment tools that have been used in slightly modified versions for the assessment of several science classes [14–17]. Briefly, questions from the original biology CLASS assessment tool [14] were either used verbatim or changed slightly, for example by changing the word “biology” or “computer science” (as in the computer science version of the CLASS assessment tool, [17]) to “bioinformatics” or “computational biology.” Other questions were adapted by adding some explanatory language to make clear that a situation was focused on the biological context of a bioinformatically approached task, such as in question 13, 24, or 28 (see S2 Supporting information for complete list of statements). In some cases, the term “biology” was used instead of bioinformatics to indicate that the ultimate goal of using bioinformatics was to understand a biological question, such as in question 8 and 10 (S2 Supporting information). Overall, questions were selected based on their previously measured effectiveness in measuring student attitude changes to learning [14,15].

CLASS surveys were originally designed to assess student progress on a novice-to-expert trajectory usually in the context of taking an introductory class in the discipline [14,17,19]. The CLASS survey here had a somewhat more limited scope in assessing the introductory bioinformatics module that was delivered as part of an advanced biology class. Participants in the module assessed here were given approximately 15 minutes to fill out the survey before the first module session and another approximate 15 minutes to fill out the postmodule survey a week after the conclusion of the last module session. The survey was administered by the instructor, but answers were collected into an envelope and anonymized by an assistant before data analysis.

### Assessment methods

#### Assessment material and analysis

To assess how students respond to the use of bioinformatics as a central requirement for data analysis in a biology class, the module started with a 31-item questionnaire plus some questions about student demographics. After completing the four-week module, students were given a postmodule questionnaire that contained the same questions as the first questionnaire (except for the part asking about demographics), plus a number of questions about student perception of the usefulness of the module for their future career. All questions from this questionnaire can be found in S2 Supporting information.

Pre- and postquestionnaires were matched by a research assistant. Responses were compared to each other before and after the module, as well as to the responses of the expert group before and after the module. Expert responses were used to determine the level of maturity in student responses before and after participating in the module. For the interpretation of the student comparison to experts, only those questions were considered for which experts reached consensus in their answers. Consensus answers were defined as those for which at least two-thirds (66.7%) of experts answered with “agree” or “strongly agree” or, alternatively, for which two-thirds answered with “disagree” or “strongly disagree.” Questions for which the expert group reached consensus are indicated in S2 Supporting information.

Questions 32 to 41 on the postmodule questionnaire were only answered by students. These questions addressed the students’ perception of the usefulness of the module. Responses to these questions were binned into three groups: agree or strongly agree, neutral, and disagree or strongly disagree. Academic transcripts (cumulative college grade reports) of students were used to determine if there was a correlation between academic strength and interest in bioinformatics. Academic strength was measured by using either cumulative grade point averages (GPAs) or GPAs only for science, technology, engineering, and math (STEM) courses. Pearson correlation values were calculated using R (version 3.3.2).

The questionnaire contained a question (question 25) that required a predefined answer and that acted as a control for filtering out responses of participants who did not read the questions carefully. This approach has been successfully used as an integral component of the classic CLASS surveys [14,17,19]. Participants whose answer to question 25 was anything other than the prescribed value were eliminated completely from the study. Of the 51 participating students and 50 participating experts, this eliminated eight students and one expert from the pool. The remaining responses were analyzed statistically using R (version 3.3.2) and Microsoft Excel (version 14.7.2, www.microsoft.com).

#### Statistics

Statistical significance was determined as follows: means between student responses pre versus post module participation were compared using a paired, two-sided *t* test. Post-hoc analysis to account for multiple testing was performed using both the Benjamini-Hochberg false discovery rate (BH) and Bonferroni correction methods, both with an alpha level of 0.05. For the interpretation of the *p*-values, only those that were below the corrected *p*-value were considered significant. Student responses were compared against expert responses either before or after student participation in the module using unpaired, two-sided *t* tests followed by BH and Bonferroni post-hoc correction as described above. Data were graphed using R (version 3.3.2).

#### Focus group discussions

One week after the module was completed, students were asked to participate in a 30-minute focus group discussion. This discussion was mostly designed to allow students to give feedback to the instructor in free form answers. Because the focus group was conducted by the author/instructor, some bias in answers is possible, therefore the focus group answers were not systematically evaluated for this paper. Some interesting and representative free text answers are provided in S3 Supporting information).

## Results

**Fig 3:**
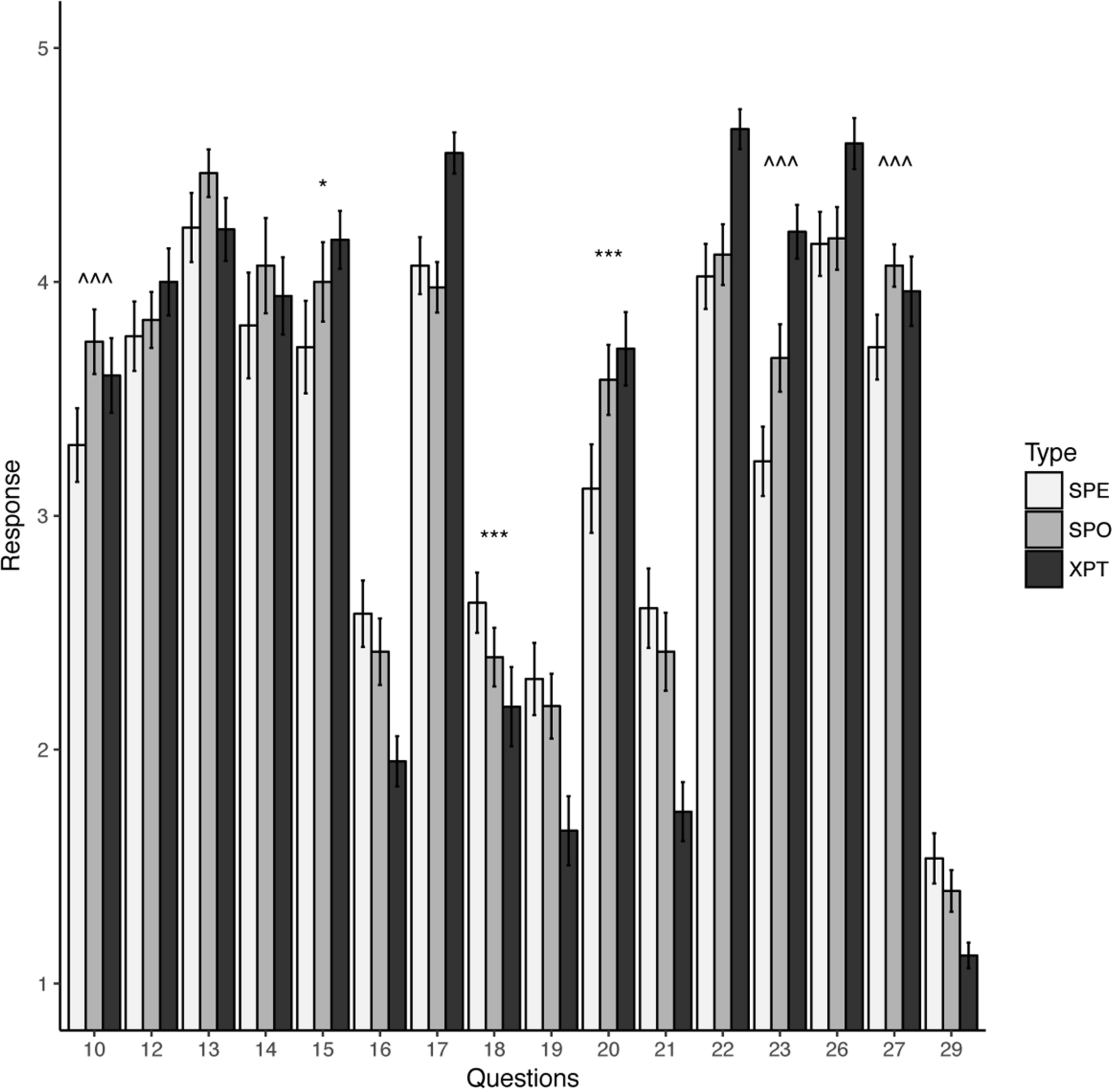
Student and expert answers to a selection of questions from the pre- and postmodule questionnaire. Student and expert responses were filtered as described in the Methods. Student responses were compared before module participation and after module participation using a paired *t* test. Student responses both pre and post module were also individually compared to expert responses using *t* tests. Error bars reflect SE. If student responses before the module compared with expert responses were statistically significantly different (at *p* < 0.05), and postmodule student responses were no longer statistically significantly different from expert responses (at *p* < 0.05), then the question was marked ***. If student responses before the module compared with expert responses were statistically significantly different (at *p* < 0.1), and postmodule student responses were no longer statistically significantly different from expert responses (at *p* < 0.1), then the question was marked *. If student responses before the module compared with student responses after the module were statistically significantly different from expert responses (at *p* < 0.05), then the question was marked ^^^. N (students) = 51; N (experts) = 50. A complete list of the questions can be found in Supporting information. SE, standard error; SPE, student response pre module; SPO, student response post module; XPT, expert response.

Questions were divided into two categories: those for which expert answers were in agreement (as defined in the Methods section) and those for which experts did not agree. Experts agreed on 18 of the 31 questions on the questionnaire. Student responses before and after module participation were compared against each other, as well as against the expert response. Across the board, student responses after the module were closer to those of the experts among questions for which experts agreed. However, only a few student responses changed from being statistically significantly different from the experts before the module to being statistically indistinguishable from expert responses (Fig 3, S1 Fig). For example, students said that after participating in the module they were more confident that they could tackle a biological question that required some bioinformatics (questions 18 and 20), and, unlike before doing the module, they now agreed with experts on the statement that understanding of some biological concepts requires quantitative skills not found among the general public (question 26).

Students responded to some questions significantly differently after the module as opposed to before the module even though their responses did not reach the same level as that of experts. In this category were questions about the importance of computer science skills and students’ willingness to try computer science to solve biological questions (questions 20 and 23). Finally, there were several questions for which experts did not find consensus, but students changed their responses statistically significantly after exposure to the module. Four of those questions dealt with the general concept of computational biology as a way to study biology (questions 4, 5, 6, and 11), and one question (question 10) was about the participant’s level of enjoyment of bioinformatics, which had increased.

**Fig 4:**
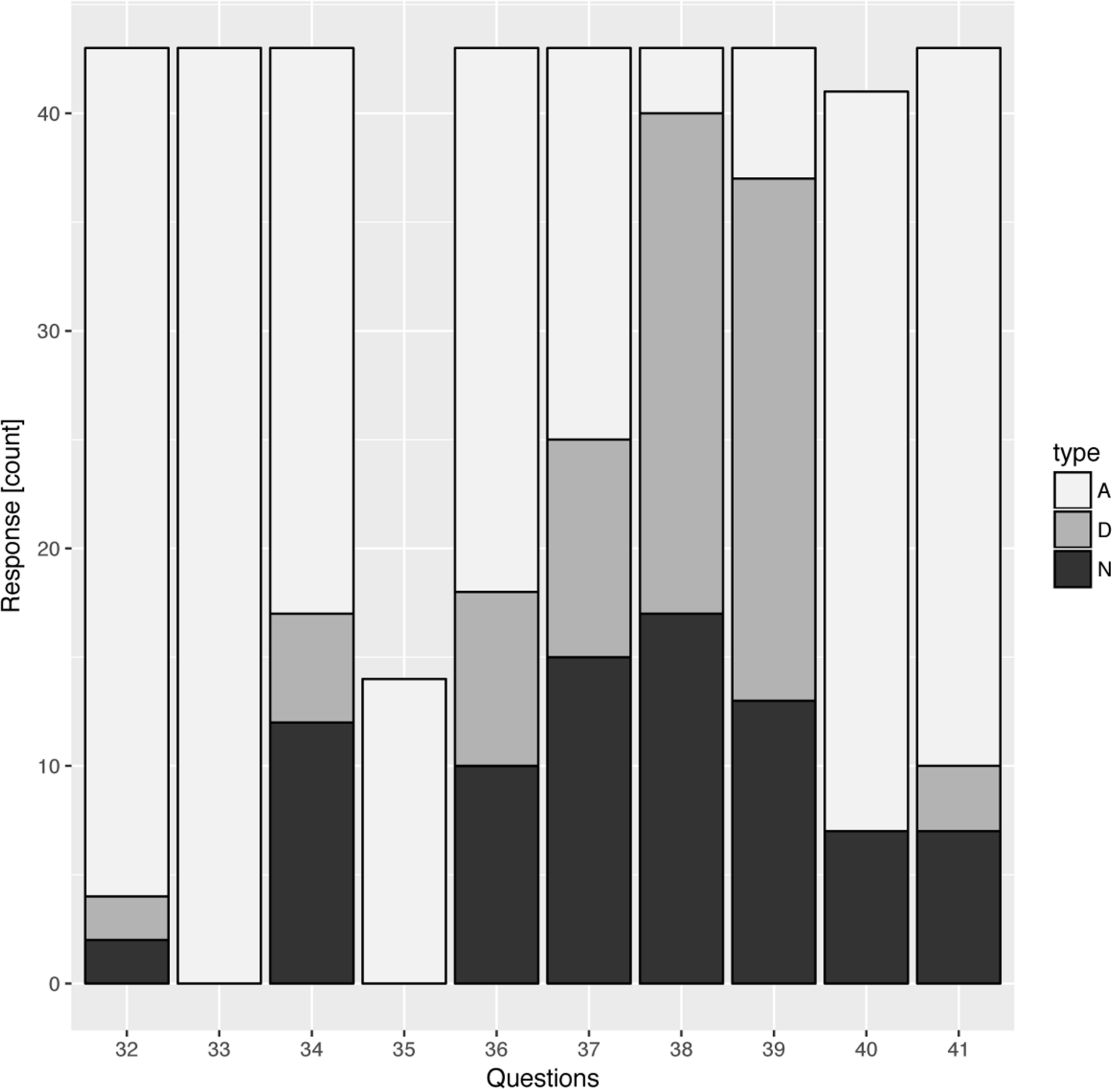
Student perceptions of the usefulness of the bioinformatics module used in this study. Student responses were binned into three groups: agree (A), disagree (D), and neutral (N). Student responses were filtered as described in the Methods. Students had overwhelmingly positive responses to their experience with the module. N (students) = 51. A complete list of the questions can be found in Supporting information.

Questions 32 to 41 asked students about their impression of the module’s usefulness and the impact it had had on them. Students stated that their curiosity about bioinformatics had increased, that the module had provided them with new insights into bioinformatics, and that they were more likely now than before to enroll in a general computer science course. Many students even stated that the bioinformatics module was “downright fun” (Fig 4).

Variation in student responses between the three different cohorts was not explicitly measured because of the relatively small size of each individual cohort. However, the overall impression from the raw data suggests that semester-to-semester differences were small.

In addition to the student participants, two biology professors also new to bioinformatics participated in this study. These professors completed the module on their own time using only the instructions of the module and requiring only minimal help from the instructor. One of these professors subsequently used the module in her own class in a slightly modified format (shorter weekly meetings distributed over the entire semester) and a smaller class of about 8 students. This professor reported that students were very engaged and provided enthusiastic feedback but did not conduct a formal assessment. The other professor reported satisfaction in her ability to learn RNAseq analysis in this self-taught way but has not yet used the module for teaching in her own classes.

To see if there was a demographic group among the students who had benefitted more from the bioinformatics module than other students, all responses to questions 32–41 were statistically correlated with the students’ general GPA, their science GPA [Biology, Chemistry, Geology, and Physics classes only), and their math/computer science GPA. Interestingly, there was no correlation between any of these answers and any of the student GPAs.

## Discussion

The call for modernizing biology education to include more bioinformatics in the undergraduate curriculum is not new [8,20,21], yet it is questionable whether or not the rate at which curricula adept at the new realities can keep pace with the speed of the development of new computational tools and methods [9,22,23].

The increasing need for life scientists to become proficient in large-scale data analysis and data management and also be able to teach these skills to undergraduates has created a bottleneck in the ability of life science faculty as a whole to keep up with the demand [24–26]. On average, lack of experience within the faculty correlates with less exposure of biology undergraduates to these techniques and creates a problem for graduates who lack an important skill set that would make them competitive in their search for jobs in industry or their applications for graduate school [22,27].

While over the last two decades the use of statistics has more and more permeated all levels and subject areas of biology instruction, bioinformatics is often still largely relegated to upper-level elective courses, although efforts have been made to introduce bioinformatics more widely in more biology classes [28]. A key obstacle for the teaching of bioinformatics in more diverse kinds and levels of classes is the lack of the required skills in teaching applied bioinformatics [24] to undergraduates in biology in more classes than just advanced elective courses, which reach only a small portion of the student population [22,23]. Formal university training of current biology teachers or college faculty very often predates the dramatic increase in the requirement for bioinformatics in the life sciences, necessitating that training of in-service faculty precede student training [9,29–31]. Finding enough expertise in bioinformatics to develop new biocomputing-intensive biology programs is particularly difficult at small liberal arts colleges [9] because many faculty received their own training before the sudden expansion of high-throughput bioinformatics-intensive research methods were developed [23], and turnover within small faculties is slow, resulting in only slow replacement of retiring faculty without bioinformatics skills with young faculty who possess these skills. While financial and practical constraints will likely prevent rapid retooling of existing faculties, it is incumbent upon universities and programs to find feasible means to speed up the process of modernizing programs to prevent opening up an ever-widening gap between what has traditionally been taught in undergraduate biology programs versus the skills that practicing biologists need now and in the future.

The choice of assessment instrument for this module allowed the analysis not only of student perception of learning and engagement before and after participation in the module but also the normalization of student responses to the expert group. CLASS analysis studies have used this approach in the past with good results [14–17]. One aspect in which this study differed from previous CLASS studies was the choice of experts. Using potential bioinformatics users who were, for the most part, not bioinformaticists by training, allowed the comparison against a group of practitioners who were likely to be more similar in their interest of using bioinformatics tools for the primary goal of analyzing biological data, not creating new tools, algorithms, or software programs. This is the group of bioinformatics users that this module was trying to reach. Given the relative heterogeneity of this expert group that reflects the diversity of life scientists from all biological disciplines, it was not too surprising that the group reached consensus on a comparatively smaller number of questions on the questionnaire than would be expected, for example when biology students in a biology CLASS study were compared with biology professors as the expert group [14]. Interestingly, one question about which the expert group did not reach consensus regarded their enjoyment of bioinformatics, indicating that skills in this field are often seen as little more than a tool to allow the scientist to analyze the data in their chosen subject area more fully.

The assessment suggested that the studied module reached some of its goals with the students (Fig 3, Fig 4, Table 1, Table 2), particularly with respect to an increased awareness of the tools available and to a gain in proficiency in their application (as shown, for example, in the change in response to questions 18 and 19), greater confidence that mastery of these tools can be learned by anyone (question 27), and a higher level of curiosity to learn more about bioinformatics in the future (questions 10, 15, and 20).

**Table 1:** Categorization of questionnaire questions by learning objective. Questions used in this survey were closely adapted from previous CLASS surveys [14–16]. Categories are as in Semsar et al. 2011. The learning objective column lists the stated learning objectives* of the module as they are addressed by the statements/questions on the survey.

Question/Statement	Learning Objective
3,4,5,7,8,17,18,19,28	A
10,14,15,20,24,27	B
3,4,5,6,12,13,16,21,23,30,31	C

*The three stated learning objectives of the module are (a) teach students basic command-line computing and bioinformatics skills, (b) motivate students to develop an interest in bioinformatics approaches to address biological questions, and (c) allow students to develop an appreciation for the use of bioinformatics in modern biology.

Abbreviation: CLASS, Colorado Learning Attitudes about Science Survey.

**Table 2:** Classification of the three learning objectives* as addressed by questions/statements on the CLASS survey.

Question/Statement	CLASS Category
1,2,8,10,12,14,15,24	Personal Interest
2,10,12,13,16,21,30	Real World Connection
3,5,18,28	Problem solving: synthesis and application
6,17	Problem solving: strategies
7,10,17,20,24,27,28	Problem solving: effort
13,20	Problem solving: reasoning
9,11,16,19,29,31	Conceptual connections
4,22,23,26	Uncategorized

*The three stated learning objectives of the module are (a) teach students basic command-line computing and bioinformatics skills, (b) motivate students to develop an interest in bioinformatics approaches to address biological questions, and (c) allow students to develop an appreciation for the use of bioinformatics in modern biology.

Abbreviation: CLASS, Colorado Learning Attitudes about Science Survey.

While the statistical analysis of answers on the questionnaire indicated that participants’ attitudes had reached parity with the experts in some areas, other areas did not allow that conclusion. In many cases, however, student responses suggested that their postmodule attitudes had shifted towards the attitudes of the expert group, just not to a degree that would make student responses statistically indistinguishable from expert responses. The most parsimonious reason for that is that a four-week module simply might not provide enough exposure for the students to reach the level of sophistication that experts have. Areas in which students most conspicuously did not improve much after participation in the module included questions about the respondents’ ability to make sense of abstract or multilayered situations, such as question 17 (“There are times I think about… a…question…in more than one way”), or about their level of problem-solving sophistication, revealed in examples such as question 3 (“…I have difficulty applying [learned] information to answer questions on similar aspects…”). Addressing these gaps would likely require longer modules, repetition, and application of learned material to more than just the one example studied in this module. Interestingly, personal interest questions, such as statement 10 (“I enjoy figuring out answers to biology questions that require bioinformatics”) received more support by postmodule participants than by premodule participants or even experts (although the difference with experts post module was not statistically significant), suggesting that students liked learning how bioinformatics can help them answer questions of interest to them and created enthusiasm immediately after their experience of success at a task that, previous to the module, seemed daunting to them. Taken together, these data suggest that students enjoy the exposure to bioinformatics but that a module of this length is not long enough to provide them with enough confidence to reach that of experts. While this may not be expected, this study seems to suggest that students might both enjoy and benefit from more, similar exercises either in the same context/class or a different class soon after their first exposure.

While there is widespread agreement that lack of adequate undergraduate bioinformatics instruction creates a disadvantage for students without access, opinions differ on the question of how to introduce biologists to computational thinking. Some argue for the integration of bioinformatics modules like the one described here into existing college classes, acknowledging that most undergraduate biology curricula are already so full that they might not allow the addition of a mandatory bioinformatics course for all biology majors [9,23,30,32,33]. Others call for the addition of new, more fundamental mathematics-focused courses for biologists [6,8] or even specialized courses that go far beyond elementary computer programing [24–26]. Indeed, some bioinformaticists have warned against the mere use (and by extension teaching) of applied bioinformatics without a deep understanding of the mathematical or computational background underlying bioinformatics analysis programs [34]. Their argument is that employing these tools as “black boxes” without a deep understanding of their underlying mathematical and computational principles can lead to misinterpretation of the results and errors in their use [34,35]. No matter which argument carries the day in any given biology department at the moment, it will be important for all undergraduate programs in biology to assess to what degree bioinformatics, and the necessary background material to understand it, will in the near future have to replace topics currently represented in typical curricula. While acknowledging the dangers discussed by May, Rubinstein, and Chor [34,35], this paper argues that it is all but inevitable that life scientists today will have to learn to use “black box” bioinformatics—at least to such a degree that life scientists can use methods and programs developed by others—and apply them to novel research questions without having to be experts in the underlying disciplines in addition to biology and their research specialty. On the other hand, in today’s world of big data, one might argue that it is incumbent upon aspiring biologists to acknowledge these rapid changes and learn the necessary underlying concepts of bioinformatics and statistics along with their applications. But change does not happen overnight. It is therefore important to empower faculty and students alike to tackle tools that may not come naturally to some practitioners in the life sciences and to engage with methods across the curricula that not only hold great promise but without a basic understanding of which biology graduates will be ill prepared for a career in the life sciences of the future.

In addition to creating a useful teaching tool introducing biology students to applied bioinformatics, a secondary goal of this study was therefore to design and assess a module that would encourage greater adoption of bioinformatics tools into non-bioinformatics college courses while at the same time allowing novice students and nonexpert teachers alike to engage in state of the art next-generation sequence analysis, learn basic bioinformatics tools in the process, and raise their interest in increased engagement with bioinformatics in the future—or at least provide appreciation of the power of these tools in life science research. While assessment of teaching faculty experiences with this module was only minimal and anecdotal, it will be both interesting and important to more formally assess faculty experiences with the use of this and similar modules. Their attitude to using modules like these in their non-bioinformatics core and specialty classes will be important to assess if the introduction of bioinformatics tools into many non–bioinformatics-oriented undergraduate classes across the curricula is to be successful.

## Supporting information

S1 Supporting information Bioinformatics module used in this study. (PDF)

S2 Supporting information Questions on student/expert questionnaire. Questions 1–31 were asked of both students and experts. Questions 32–41 were only asked of students after completing the module. Questions for which experts achieved “consensus” (more than 66.7% of answers were answered either as 1 or 2, or as 4 or 5) are italicized.
(PDF)

S3 Supporting information Focus group questions and examples of student free text responses about their experience with the module. These responses were recorded from students during a focus group analysis one week after the conclusion of the module. Questions were asked in seven separately conducted sessions using a standardized script.
(PDF)

S1 Fig Student and expert responses to all questions on the questionnaire. N (students) = 51; N (experts) = 50. Unless reported in Fig 3 (which shows a subset of these data), student responses compared with those of the experts did not differ between pre- and postmodule testing. This figure shows responses to all questions, including those for which experts did not reach consensus (see Methods and Results). SPE, student response pre module; SPO, student response post module; XPT, expert response.
(PDF)
