# Supplementary material for: Assessing an effective undergraduate module teaching applied bioinformatics to biology students
Source: PLoS Comput Biol. 2018 Jan 11;14(1):e1005872. doi: 10.1371/journal.pcbi.1005872 (PMC5764237; doi:10.1371/journal.pcbi.1005872)
Supplement: S1 Supporting information — (PDF) [file pcbi.1005872.s001.pdf]

**S1 Supplemental Material**

# Bioinformatics lab: Introduction to command line computing with CyVerse/iPlant using the Tuxedo pipeline for RNA-seq (lab 1)
## Introduction to Unix

This exercise will introduce you to some simple Unix commands that you will need later to work with data files. At the beginning, Unix will seem to be cumbersome and annoying to you. Over time, commands will become second nature to you and you will start to appreciate the power of Unix.

## PRELAB (5 points)

Please sign up for an CyVerse/iPlant account **AND** request access to "Atmosphere" **AND** launch an "instance", **AND** suspend it as follows:
Note that depending on your access rights and website updates, some of these website URLs might change. In that case, follow the latest instructions on the CyVerse and Atmosphere websites to create an account and gain access to Atmosphere. CyVerse is the new name of iPlant. You might find references to both in this module. Both refer to the same computing platform.

**Signing up for CyVerse**

1. Go to http://www.cyverse.org/learning-center/create-account and sign up for a free account.
2. After signing up, request free access to "Atmosphere". https://user.cyverse.org/services/mine . It may take a few days for your account to be set up and access to Atmosphere granted.

**Logging in, launching an instance**:

1. If you logged out since creating the account, go to http://www.cyverse.org/learning-center/ and login using your user account and password.
2. Login to Atmosphere.
3. Click on Launch New Instance. In the image search box type the name of the image you will use for RNAseq analysis: **RNAseq_Analysis_gt_V2**.
4. Click on Launch New Instance. In the image search box type the name of the image you will use for RNAseq analysis: **RNAseq_Analysis_gt_V2**.
5. Select this image, click Launch, select a "large 3" instance from the pull-down menu, leave the other options at their default, and click "launch instance".
6. Sometimes instances get stuck during deplaoyment. To get them unstuck, try redeploying or rebooting (hard reboot) them using the buttons on the right of the "Projects" tab.

Starting the instance may take up to 30 minutes but sometimes it takes just a few minutes. You will receive an email to your email address that you linked to the account telling you that the instance is ready and providing you your IP address and the ssh username. You will need both in the next steps.

4. When your instance is launched you are ready for data analysis. For right now, however, click on “suspend” instance. Wait a few minutes for it to suspend, then log out and close the website. You are now ready to quickly get started when you come to lab, because waking up a suspended instance is much quicker than launching a new one.

# Week 1: In-Lab Exercise 1

For the following exercises your do not need access to iPlant. A Mac computer is preferred. If you do not have a Mac, use an iPlant instance instead.

**Exercise 1: Working in the Unix/Linux environment.**

1. Go to the Terminal program (or your emulator if you are using a PC) and open a Terminal window.
2. Your window opens and shows you the owner and user names followed by a $ sign, as in:
   TH223F-6657:~ amadlung$
   Every computer has a specific name plus the login name of the user and will be different for each of you. Commands you enter will be entered after the $. You do not type the $ in your commands. Later on we will do all of our computations on a remote server, to which you have to log in. You can then use your keyboard and screen to run that remote computer/server, using Terminal.
3. For now let's learn some very basic commands to be able to move around on your own computer. Following you will see in this document exactly what you have to type after the $ sign. Also, anything following a # is NOT entered, and only serves the purpose of explaining parts of the command to you.
4. Remember from the lecture that each command is composed of multiple parts: the command itself, any options (specifics for the command), and the argument (usually the file you want the command to be worked on). Start by typing in this simple command and hit return.
   a. `$ ls` # After the $ sign type ls and hit return. (You never have to type the $ sign. It automatically shows up in an active command line.) This will list all the files that you have in your home folder. Note that there is no option and no argument.
   b. Let's augment the command with an option. First use `$ls –a`
   c. This command lists all files, even those normally hidden to regular users. Now type `$ls –lh`
   d. The list of files includes so called "permissions" (the dwrx--- etc string), date, the file size, and a few other things.
   e. Now type `$ls-lh` but leave out the space between ls and –lh. You will see that you get an error. One thing to note with Unix is that it only recognizes exactly what you type and will not second-guess you. If the command is not typed correctly, Unix will not do anything for you. Likewise, please be careful because Unix does not ask you if you are sure about what you are asking the computer to do. For example, if you use a command that directs Unix to delete your hard drive, **Unix will do so without warning**.
5. The next thing to learn is how to move around on your computer to access or specify documents. The unique address of a document on your computer is called ***the path***. You are probably used to windows, using a mouse, and dragging-and-dropping. Unix requires written commands for everything.

Understanding the hierarchy of the file system is very important, or else you are moving around in the dark.

Unix calls folders “directories” and files “documents”. In this figure a directory located inside another directory is designated with a line between directories. The root directory is on the left marked with a slash /.
Unix computers have a “home directory” (in the example figure above called “lucy”). Generally, data files you work with should be stored downstream of the home directory, for example the desktop or in Document directories. Upstream of your home are directories that contain software programs, such as Unix. An important one to know is the bin directory.

Go back to your terminal window. We will now move around in the file hierarchy. You should be in the equivalent location to `TH223F-6657:~ amadlung$`, which is your home directory. To verify your location you can use the command `pwd` (print working directory). Try out the following commands:

a. `$pwd` #You should now see /Users/amadlung (or in your case your username). Look back at the figure and notice that the home directory is in the Users directory. Now let’s move down one step in the hierarchy and enter the Desktop folder. The command cd (change directory followed by your destination) will do that.
b. `$ cd Desktop` # Make sure to use the correct spaces. After the $ there is a space and after cd there is a space. Note that with cd you can only go to a directory that is **one step removed** from your current location. If you want to jump into a directory elsewhere, you need to specify the entire path. We will learn how to do that soon. For now look at the content of your Desktop like this:
c. `$ ls` # With this command you are listing all the folders and files on your Desktop. You are now “inside” that directory and you cannot get ahold of any file or folder outside this directory. You can either work on a file in the directory you are currently in (Desktop) or you can move deeper into the file structure (in some cases aka a complete unorganized mess) or you can move back out into your home directory. To move backwards in the hierarchy, use the command cd space dot dot (cd ..). This moves you to the *parent directory*. Try this:
d. `$ cd ..` # Use cd followed by a space and two periods (followed by hitting return) and you are now back in your home directory. To convince yourself that you have returned to the home directory you can use a `ls` command and look around or you can use `pwd` to ask where you are.
e. You can get back to the home directory from anywhere (not just down one directory) using `cd ~`

6. Next you should learn a very useful trick that avoids misspelling files or directories and also allows you to see if the computer finds the file or directory you specify as you are putting in a new command. This can help you avoid frustration when your computer refuses to do what you want (since it often won't tell you exactly why it refuses a command).
    a. Go to your home directory and type in Desk as if you were typing Desktop. Don't complete the name yet and after Desk hit the tab key. Like this: `$ cd Desk`    #Then hit TAB. You should see that the computer "autocompletes" the file or directory name to Desktop/ Make it a habit to use autocomplete all the time. If you don't have the correct file in the correct directory or you are misspelling it, autocomplete will not work and you will know that you have to fix something.
7. Next, let's learn what the *path* to a file is. Assume you are lost in your file structure. One way to find out is to ask the computer where you currently are, using `pwd`, as you learned above.
    a. `$ pwd`    #The "present working directory" command returns your location. If you are not in your Desktop directory, move into it now. Then type: `$ pwd`
       The computer will tell you more than you expected: It will give you what is called the "file path". Usually that starts with the *Users* folder followed by the username (your username, which is the name of your home directory), followed by the directory you are in. Right now you should be in the Desktop directory, so you should see this: /Users/yourusername/Desktop #where yourusername is in fact your actual user name (not "yourusername", which I am using here as a place holder).

8. There are two ways to use the *path*. You can specify a file on your computer by typing in the entire list of hierarchical directories from your home down to the file you want to specify. For example /Users/yourusername/Desktop/yourfile would be one way to specify the file. The file path is difficult to understand for a new Unix/Linux user.

    The file path is the only way to specify a file to the computer to work on. There is the "**absolute path**", which lists all directories from the "Users" directory on down to where your file is located (like a Russian doll), or you can use shortcuts - if you understand them. As you start out using Unix, make it a habit to always move into the folder in which the file is located that you want to use for a command.

**DO THIS:** <u>Draw a partial hierarchical tree structure similar to the figure above but indicating some of the directory names actually on your computer.</u>
Draw the file system of YOUR computer on a large piece of paper. Use the commands `cd, ls,` and `cd ..` to move around the computer. After each step up or down,

use `ls` to list the folders on your system. (No need to draw all of them, just the ones similar to those in the figure but with your names).
**As you progress through the exercise you will create more directories on the desktop and later inside the new directory on the desktop. Add those directories to your tree.**
**TURN IN THE DRAWING OF THE TREE AT THE END OF LAB.**

**Exercise 2: Making files and directories, and using wild cards**

1. In Terminal, go to your Desktop directory using the command `cd` Desktop.
2. Using command `ls`, list the files on your Desktop.
3. Now create a new file by using the command `touch` to create a file called:
   Bio332_firstfile.txt
4. Use `ls` to list the files in the directory. Your Desktop directory should now list the file you just created. Verify that this is true.
5. Use touch and create a second file called Bio332_secondfile.txt.
6. When you use autocomplete to search for a file, the computer might detect similarly named files. In this case it will autocomplete up to the first ambiguous letter of the file. We will try this out in the next step with your two Bio332_ files where we will remove one of the files.
7. To delete a file use the command `rm`. Type Bio4 and then hit tab to autocomplete. You will hear a warning signal and the computer will autocomplete only as far as Bio332_. Type in f, which is the next letter that will resolve the ambiguity between the two files Bio332_firstfile.txt and Bio332_secondfile.txt. Hit tab to autocomplete. The rest of the file name should fill in automatically. Hit return and Bio332_firstfile.txt is deleted.
8. Use `ls` to verify that the file has been removed.
9. Ok, you now know how to delete a file, but it turns out that we do need this file later, so remake the file Bio332_firstfile now.
10. Next we will create a folder/directory. Use the command `mkdir` to create a folder called Bio332_unix_data on your desktop. Use `ls` to verify that you have the folder.
11. Next we will move the file Bio332_firstfile.txt into the new folder using the following command:
    `$ mv Bio332_firstfile.txt Bio332_unix_data/` #Notice the command (mv) and the argument (your file) followed by the place where to put the file.
12. Now move into the unix_data directory using the `cd` command. Once in the correct folder, use `ls` to verify that it contains Bio332_firstfile.txt.
13. Move back into the Desktop directory and move Bio332_secondfile.txt into the Bio332_unix_data directory. To move back down one level in the hierarchy use `cd ..`
14. Next we will learn the use of wild cards, which can help you later save a lot of time. Wild cards are used to list ALL files that have certain parts of their file name in common. First, create a new directory inside your Bio332_unix_data directory and call it Bio332_unix_data_2ndlevel.

15. Use ls to list the content of your Bio332_unix_data directory. You should now have two files and a directory inside this directory.
16. Use `ls *.txt` to list all files that end in .txt.
17. Now move your two .txt files into the 2nd level folder by using the following wildcard command: `mv *.txt  Bio332_unix_data_2ndlevel`. Use autocomplete to avoid (mis-)typing the whole directory name.
18. Use `cd` to move into the 2nd level directory, then use `ls` to check if you moved the files. Then use `cd` to move back one level and check if your directory Bio332_unix_data is empty as it should after moving the files to Bio332_unix_data_2ndlevel.

**Exercise 3: Downloading data from the web using Unix**

1. Later we will load data into your Bio332_unix_data directory that we can work with. Much of the data we use will be downloaded from FTP sites. The first data we will download is simply a copy of the Unix commands cheat sheet from this website:
   ftp://ftp.solgenomics.net/bioinfo_class/other/interns/
   Alternatively you can try: ftp://lxftp01.pugetsound.edu/pub/

   Using a regular web browser, open this page, locate the pdf file unix_command_sheet.pdf and copy the "link location" into the computer's memory. There are multiple ways to do this: Either control-click, or right-click, or tap with two fingers on the file. From the drop down menu that will appear select "Copy link location".
2. Go back to the Terminal and use the command `curl` (stands for copy URL) as follows:
   `$ curl –O` (this is an Oh, not a zero) add one space and then paste the link with command-v into the terminal window.
   (The –O option following the command `curl` creates a file with the same name as the one you are downloading via FTP.) So the full command is
   `$ curl –O ftp://ftp.solgenomics.net/bioinfo_class/other/interns/unix_command_sheet.pdf`   # There is a space between the command and the ftp address.
   (If you use the terminal window in iPlant and not your own computer, the command is wget, not curl.)
3. Check your success with `ls`.

**Exercise 4: Using a text editor**

Next, we will create a file, write text in the file, save the file, read the file, and eventually move files around. Earlier, you created and deleted a file on the desktop. Create a new file using `touch` in the directory Bio332_unix_data_2ndlevel and call it "Bio332_data_file1" (not typing the "  "). Use `ls` to verify.

1. To write things in a file you have to use a "text editor". There are many options available. We will use one that is already loaded in Terminal called emacs (one loaded in PC terminals is called nano). On the command line type $ `emacs Bio332_data_file1`
2. A blank window will open. Now type the following text in the window: "This is the test file text".
3. To save and close the editor use control-X followed by control-S (This command means holding both the control and the X (or S) key at the same time.) Then use control-X followed by control-C to close the editor.
4. To open a file and read the content you can use the text editor or use a unix command called `less`. Type $ `less Bio332_data_file1`. Use autocomplete instead of typing the full name!
5. To close the file use `q`. (Note that using a text editor and using less and q are different ways to read a file. Only if you use the editor can you actually edit the file, though.)
6. Next we will move the file. Make sure you are in the directory Bio332_unix_data. Make a new directory called "Bio332_unix_subdirectory1" (no quotes and don't forget the underscore between the words) into which we will move the file. This new directory should be inside the current directory (Bio332_unix_data). Use `ls` to verify.
7. Move yourself into the new directory to see if you created a directory as opposed to a file, into which you cannot move (which command do you need?). Move back out to Bio332_unix_data.
8. You should be back in the directory Bio332_unix_data. To move a file you should be in the directory *from which* you want to move the file. Now move the .pdf file you have in the Bio332_unix_data directory into the Bio332_unix_subdirectory1 using the following command:
   `$ mv unix_command_sheet.pdf Bio332_unix_subdirectory1/`
   #make sure there is a space between mv and the file name and the destination directory.
9. Move into the directory Bio332_unix_subdirectory1 and verify that the .pdf file was moved there and is now also gone from the unix_data directory.

**Exercise 5: Writing scripts, changing permissions**

1. We will now download data files into the Bio332_unix_subdirectory1 directory. If you can, use a hard-
   wired internet connection (not wi-fi) to speed up the process considerably.
2. Go back to your web browser and browse to ftp://ftp.solgenomics.net/bioinfo_class/interns/
3. From there drill down five levels as follows: data → ch04 → breaker → SRR404334 → SRR404334_ch4.fq
4. (If steps 2+3 don't work go to http://solgenomics.net/ and click on 'tools' in their menu bar and select 'FTP Site.' Click on 'FTP Top Level' and from here → bioinfo_class → other → interns → data → ch04 → breaker → SRR404334 → SRR404334_ch4.fq)

5. Right click (or two finger click) on SRR404334_ch4.fq to copy the link location. Then download the file into the Bio332_unix_subdirectory1 using curl –O
6. `curl -O` ftp://ftp.solgenomics.net/bioinfo_class/other/interns/data/ch04/breaker/SRR404334/SRR404334_ch4.fq # There is a space between the command and the ftp address.
7. This will take a while. The file is quite large (73.8MB).
8. Use ls to verify it is in the correct folder Bio332_unix_subdirectory1.
9. Type `$ head SRR404334_ch4.fq` (use autocomplete) and hit return. This command returns the head of the file, which is just as much as it can fit onto your screen. Never mind what it says for now.
10. Now try `$ less SRR404334_ch4.fq` (use autocomplete). The less command allows you to scroll through the entire file window by window. The first window is displayed. Hit the space bar and you the next section is loaded or use up and down arrows to scroll. To get out of the window use `q`.
11. Use `tail` to view the last bit of the file. (This is often useful to see where the file ends, if the file is complete, or if data manipulation that you have performed finished all the way to the end.) `tail SRR404334_ch4.fq`
12. We need the other three files that are similar to the one we just downloaded. Doing the same task 4 times is tedious, so let's write a small script that will do the work without us having to perform individual downloads. The type of script you will write is called a shell script and has the file extension .sh (for "shell script").
13. Create a new file using `touch` and call it Bio332_script_1.sh
14. Use `ls` to check, but use the option `–l` (this is the letter l, not the number 1) with your command to see the list of your files, including the permissions. (Remember to leave a space between command and options). You should get something like this:

    ```
    -rw-r--r--  1 amadlung  staff  0 Sep 14 09:48 Bio332_script_1.sh
    ```

    The dashes and letters x (execute), r (read), and w (write) list the type of permissions associated with the file. (This one currently only has r and w permissions.) The first character can be a dash (for a file), letter d (a directory) or letter l (link). The next three characters (2-4) are the permissions for the *owner* (that is you), the next three (5-7) for the *group* (that depends on the network configuration, but could be all of a small group using the same server), and the last three (8-10) are for *other*, which is everyone getting access to this script. Usually you want to be able to execute a file you write (that is its purpose) and often want to allow co-workers to use it too, but not allow access to change things by just anyone. The types of permissions are coded using numbers: 4 means "give permission to read", 2 means "give permission to write", 1 means "give permission to execute". In the command option these numbers are added up for each of the three "modes" (read, write, execute). Therefore a 7 in the first digit would mean

4+2+1 = give read, write and execute permission to the owner. A 5 in the second digit would mean give permission to read and execute to the group. And a 5 in the last digit would mean the same but for everyone else. So using the code 755 means to give read and execute rights to all but only you can write/edit the script.

| | | | |
|---|---|---|---|
| 7 | 5 | 5 | |
| user | group | everyone | |
| rwx | rx | rx | |
| 4+2+1 | 4+0+1 | 4+0+1 | = 755 |

15. Use `$ chmod 755 Bio332_script_1.sh`, then use `ls –l` to check if the permissions were changed as you intended.
16. You now have an executable script, but no commands in the script yet. Next we will write the script text. Using the [ftp.solgenomics.net](ftp.solgenomics.net) website you have used above, put four lines of code into the script, each separated by a return/enter, that use `curl –O` and the ftp address of the four files you need (two from the "breaker" directory, and two from the "immature fruit" directory). In the end your script should look like this:

```
curl -O ftp://ftp.solgenomics.net/bioinfo_class/other/interns/data/ch04/breaker/SRR404336/SRR404336_ch4.fq
curl -O ftp://ftp.solgenomics.net/bioinfo_class/other/interns/data/ch04/breaker/SRR404334/SRR404334_ch4.fq
curl -O ftp://ftp.solgenomics.net/bioinfo_class/other/interns/data/ch04/immature_fruit/SRR404331/SRR404331_ch4.fq
curl -O ftp://ftp.solgenomics.net/bioinfo_class/other/interns/data/ch04/immature_fruit/SRR404333/SRR404333_ch4.fq
```

17. Since you already have the *334_ch4.fq file you can delete it from your script to save time during the download.
18. Use control-X (hold both keys down together starting with the control key) followed by control-S, and then control-X followed by control-C to save and close the script file.
19. To run the script all you have to do is to navigate into the directory where the script is, then use `./Bio332_script_1.sh` (use autocomplete). Note that there is NO space between the ./ and the script name.
20. The script will now download your three files specified in the script. The Terminal window will show you the progress in real time. When done, Terminal will return to the command line. Use `ls –l` to check.

**Exercise 6: Using screen, uncompressing and concatenating files, pattern search with grep**

1. Create a script called Bio332_script_2.sh. Change the permissions to allow you to run it. Using the text editor emacs, write the script so that it will fetch the file called [unix_class_file_samples.zip](unix_class_file_samples.zip) from the website [ftp://ftp.solgenomics.net/bioinfo_class/other/interns/](ftp://ftp.solgenomics.net/bioinfo_class/other/interns/) . Save the script.
2. This time we will run the script a different way. When downloading large data files there is always a chance that the connection from your computer to the server you are using is interrupted momentarily. This leads to a "break in the pipe" and incomplete file downloads. To prevent that the command from being interrupted, you can use a command called "screen". When using screen the command runs in the background, independent of your computer.

For running scripts or commands, using screen is the best option to avoid time-consuming server problems. Run your script like this: `$ screen ./Bio332_script_2.sh` Note that there is no space between ./ and the script file name.

3. Use `ls –l` to check. You should now have a .zip file in your directory. The zip file contains several compressed files that we will have to uncompress now. Use `$ unzip unix_class_file_samples.zip` # Note that there is exactly one space between $ and the command "unzip". Use `ls –l` to list the new files that expanded into your directory.
4. You will notice that you have three files called sample1/2/3.fasta and three more files called sample1/2/3.fastq. We will now merge the three files into one file that contains all the sequences that are in each of the files. We will make one file for the .fasta files and one containing all the .fastq files. To do this for the first file we can use the `cat` command, list the three files we want to merge, and the > sign to designate the new file name. Instead of typing out three file names, let's use the wildcard *fasta. Before you start doing stuff with a wildcard, make it a habit to always first list what the wildcard will specify so that there aren't files that you didn't see that would get used as well. Use ls *fasta to list all fasta files.
5. These three are indeed the correct ones to merge, so the wildcard works for you here. Use `cat *.fasta > samples123.fasta` to merge the files into the new file named samples123.fasta. Check with `ls` and open the file with `less` to see what's in it. If you see stuff, that's good. Close with `q`.
6. Now merge the .fastq files into a new file and call it samples123.fastq
7. Open the samples123.fasta file with `less`. This file contains several protein sequences from the Arabidopsis thaliana genome. Each gene has an identifier name that starts with Atha_, followed by the gene name AT (for Arabidopsis thaliana, a number (the chromosome number the gene resides on), G (for nuclear genome there could also be a C or M for the chloroplast or mitochondrial genome), and a 5-digit number, followed by the version number, e.g. .1. All FASTA files start with a > sign. Use the space bar to step through the file. You will see it only has a few protein sequences. Let's extract the gene names only from the file and eventually put the gene names into a new file. Here we can use the pattern search command `grep`. Grep will look for a string you specify between ' ' marks. It will also take wildcards. Let's start with extracting the first gene name only: >Atha_AT1G51370.2
8. Use `grep '>Atha_AT1G51370.2' samples123.fasta`. Note the placement of the single quotes and the spaces between command, argument, and file name.
9. Unix displays the extracted phrase on the screen. If you want to put it into a new file you can specify the file name in the same command.

```
grep '>Atha_AT1G51370.2' samples123.fasta > Bio332_firstgrep
```

10. Check with `ls` and `less` that this worked out for you. Then use `rm` to remove the file. Again check that the file is gone.

11. Now use `grep` and a wildcard command (try to figure this one out yourself) to extract all gene names from the samples123.fasta file and place them into a new file called Bio332_genenames. Use `ls` and `less` to check. How many gene names were extracted and placed into the file? (If you need help with the wildcard, please ask!)
12. Another way to count lines automatically is to use the grep command with an option. Try `grep -c '>Atha_AT*' samples123.fasta` (Note that this command does not use the file name or else your file will be overwritten with the new results.)

This concludes the exercise. Make sure that all files that you made today are in the correct directories with the correct names so I can find them when I check your accounts and assign the points you earned for the lab.

## Bio 332
## Bioinformatics lab: Introduction to command line computing with iPlant using the Tuxedo pipeline for RNA-seq (lab 2)

# Prelab assignment:

1. Download the reading for this lab from Moodle (Trapnell et al., 2012, Differential gene and transcript expression analysis of RNA-seq experiments with TopHat and CufflinksNature Protocols, 7, 562-578). Don't get bogged down in details and try in your reading to get an overview of what each of the major steps accomplishes during the analysis.

2. Considering the experimental set up for the experiment whose data we are analyzing and the overall experimental question, please answer the following question by writing a 4-5 sentence paragraph and including 2-3 primary references to back up your assertions and predictions:

**PRE LAB QUESTION**: In general terms describe the metabolic pathways and reactions that occur during ripening in "climacteric fruit", such as tomato. Suggest specific genes whose expression level you would expect to change during the ripening process, explain why you think these genes would change, and give citations that would support your predictions. (Tip: see Taiz + Zeiger textbook, p 665-662)

**When you come to lab:**

**Log in, resume your running, but suspended, instance**:

1. If you logged out since creating the account, go to http://www.cyverse.org/learning-center/ and login using your user account and password.
2. Login to Atmosphere.
3. The following steps were probably already done by you previously. Only if not, follow steps 4 -6.
4. Click on Launch New Instance. In the image search box type the name of the image you will use for RNAseq analysis: **RNAseq_Analysis_gt_V2**.
5. Select this image, click Launch, select a "large 3" instance from the pull-down menu, leave the other options at their default, and click "launch instance".
6. Sometimes instances get stuck during deplaoyment. To get them unstuck, try redeploying or rebooting (hard reboot) them using the buttons on the right of the "Projects" tab.

**ONCE THE INSTANCE HAS BEEN RESUMED:**

A Mac is preferred for the following work but a PC can be used as well with a few changes (not described in the lab module, though).
**PC users only:** Go to the Start button (bottom left) and find the "search" box, which is just one row above the Start button. Type in `cmd.exe`
Locate the file cmd.exe, click on it and a black window will open in your screen that works similarly to a Mac Terminal. Use the instructions for Mac users from there.
Alternatively, use the browser as a Terminal by clicking on "Open Webshell" in the Projects tab. A shell will open. You might have to click on the X in the top right corner and/or the icon in the top middle of the shell to get the login prompt.

**Mac users:** Open a terminal window and login to the Atmosphere cloud using the unix command ssh, your username and the IP address you were sent to your email. In the example johntester is the username.

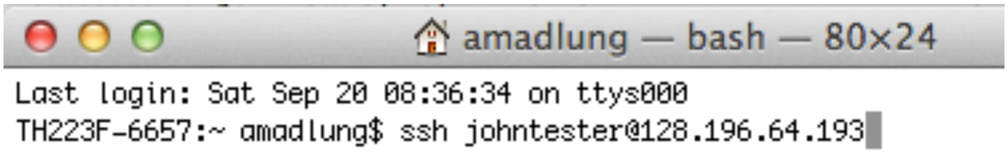


Answer "yes" when asked about wanting to connect. Then provide your CyVerse password.
You will be logged in and your window should look similar to this:

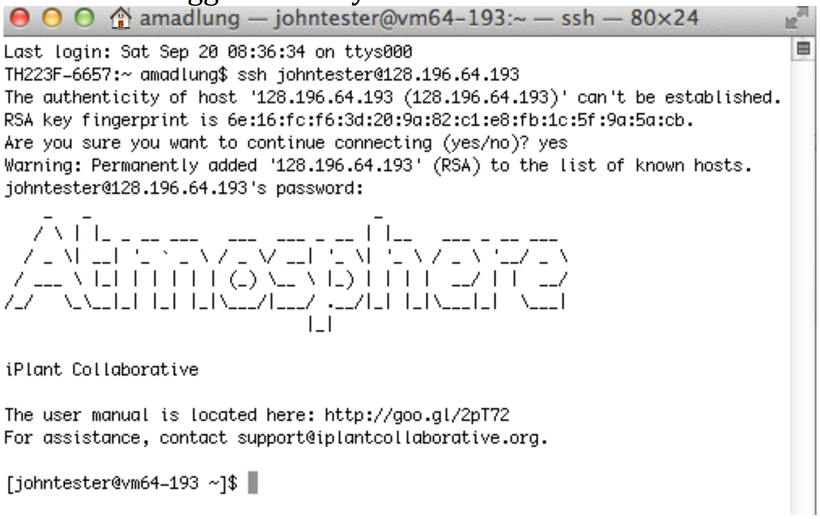


Refresh your Unix skills before continuing on by looking around in your file hierarchy. It will be vitally important that you pay attention to where in the file hierarchy on your cloud computer you create files. If you create your file for example in your home directory and later try to run a command from within your Desktop, your will get error message. After ascertaining where you are (use `pwd` if you are unsure) do the following:

1. Move to the Desktop directory.
2. On the desktop, create a new file that you call Bio332_Atmospherefile1

3. Using the text editor emacs open the file you created and type in “test text here”. Save and close the text editor. (If you don’t remember how to do that, look in the manual for the unix intro lab.)
4. Open the file with `less` to check. Then show your instructor the file with content before moving on.

Your command line history should look something like this now:

```
[johntester@vm64-193 ~]$ ls
Desktop
[johntester@vm64-193 ~]$ cd Desktop/
[johntester@vm64-193 Desktop]$ ls
idrop.desktop
[johntester@vm64-193 Desktop]$ touch Bio332_14_Atmospherefile1
[johntester@vm64-193 Desktop]$ ls
Bio332_14_Atmospherefile1  idrop.desktop
[johntester@vm64-193 Desktop]$ emacs Bio332_14_Atmospherefile1
[johntester@vm64-193 Desktop]$ less Bio332_14_Atmospherefile1
[johntester@vm64-193 Desktop]$
```

Next we need to download the four files with the RNAseq data we used last time (breaker, immature fruit) to your virtual cloud hard drive. The connection between Atmosphere and the data server is very fast, so we can simply download them from the server again (rather than upload them from the computer to which you downloaded them last time).
The iPlant server uses a slightly different unix/linux version from the one you have used so far but the only thing we need to change is using a different command to download. Instead of using `curl` we will now use `wget`.
If necessary look back at the exercise we did previously to do the following steps.

1. Make a directory on your iPlant desktop called *Bio332_BTI_exercise_data*. NOTE: double check your spelling on “exercise” - you will later run into problems if this isn’t spelled correctly.
2. Write a script to download the four files called *Bio332_iPlant_Script1*. Make sure this script is in your *Bio332_BTI_exercise_data* file, or the script won’t run without using an absolute path later on. You can copy + paste the addresses from the lab manual from last week to speed up the script writing.
3. Make the script executable by changing the permissions.
4. Download the files using the `screen` command to invoke your download script.
5. If necessary, move your just downloaded SSR* files into the *Bio332_BTI_exercise_data* directory, and check successful transfer using `ls`. Show your instructor the directory when done with this step.

**<u>RNA-seq analysis</u>**

**Step 1: Read quality analysis using the software program FastQC**

FastQC is freeware that checks the reads that come from the sequencing lab for quality. The software is available for free download from the bioinformatics group that has developed it. We will download a zipped version of the program, unzip it, install it on our iPlant instance, check the permissions of the software and change

them if necessary, move out of the downloaded folder back into our data-containing folder, write a shell script for fastqc analysis in one step, and then run the analysis. After that the analysis files will be viewed on a web browser.

1. Download FastQC: Go to www.bioinformatics.babraham.ac.uk
2. Click on "Projects", find "FastQC" and click on that, then click on "Download now".
3. Copy the link location (right click, two-finger click, or control-click) for FastQC v0.11.4 (Win/Linux zip file) (regardless of Mac or PC use, since we want the Linux version) and use `wget` to download the file into your *Bio332_BTI_exercise_data* directory.

```
[johntester@172 BTI_exercise_data]
$ wget http://www.bioinformatics.babraham.ac.uk/projects/fastqc/fastqc_v0.11.4.zip
```

4. Use `[johntester@172 Bio332_BTI_exercise_data]$ ls` to check if the fastqc_v0.11.4.zip file is present.
5. Unzip the downloaded program directory: `$ unzip fastqc_v0.11.4.zip`
6. Your *Bio332_BTI_exercise_data* directory should now contain a *FastQC* directory. Move into that directory using `cd`.
7. List the files using the option `–l` to see the permissions for the executable program fastqc.
8. If your fastqc has the permissions -rw-r--r—1 you do not have execution permissions. Since you now own the program you can change the permissions using `$ chmod 755 fastqc`.
9. Check your new permissions with `ls –l`.
10. Since you are in the *FastQC* directory, move back one level into the *Bio332_BTI_exercise_data* directory.
11. Using touch, create a shell script and call it *Bio332_fastqc_analysis.sh*. Change the permissions to allow yourself and others execution permission. Use the emacs text editor to write four lines of code, each using fastqc to analyze one of your four SRR… files. Since you are in the directory with your SRR… files this is a tiny bit tricky, because you have to specify where the fastqc program is located using absolute path notation. To get you started, the first line (of four) should look like this: `FastQC/fastqc SRR404336_ch4.fq`
12. After you have all four lines coded, save the file, close the text editor, and run the script, using screen. Take note that when using the screen command you have to
    a. be in the directory that contains the data files you want to use (in this case *Bio332_BTI_exercise_data*)
    b. follow the `screen` command with a space and then `./` followed without space by the script file name.
    → So in this case `screen ./Bio332_fastqc_analysis.sh`
13. Next you have to download the fastqc.zip files that were generated in the analysis to your own computer's hard drive before viewing them in your web browser. Open a **new** Terminal window so you can control download to your actual (not virtual iPlant) machine.
14. Using this new terminal window, customize the following command so it fits your username and IP address: `$ scp`

```
johntester@128.196.64.174:/home/johntester/Desktop/
Bio332_BTI_exercise_data/SRR404331_ch4_fastqc.zip .
```

Make sure to use your IP address, and after home/ again your username. (If you are not using your computer you can use `pwd` to see the full path to the username of the machine you are using.) There should be **no space** after Desktop/ . Note that the **space dot** after fastqc.zip is part of the command. This specifies the location on your computer. If you are prompted to enter your password, enter the password of your iPlant account. The file should be directed in your home directory now.

15. Find the file in the home directory of your actual computer (not the virtual machine), (if you are using a Mac, open a new Finder window and look in your home directory) double click on it, let it unzip, open the folder it creates, and double click on the fastqc_report.html. A browser window will open.
16. You will need to download the other three .zip fastqc result files. You could do this three more times as you did above, but you could also have done this in one single command, without even having to write a script and instead using a wildcard-containing command. Modify the command in step 14 now in such a way that you can download the rest of the files. (The file you already downloaded can be overwritten if you re-download it again – which is fine.) Make sure that you have the correct IP address and username.
17. Now go to your home directory window (not using the Terminal but the mouse), locate the four zipped files, unzip them by double-clicking, open the folders, then open each fastqc_report.html to display them on a web browser.
18. Before looking at your reports, point your web browser to https://www.youtube.com/watch?v=bz93ReOv87Y and listen to the explanations of how to read the results. (Please plug in a headset or take your computer out into the hallway.)
19. Now check your results. You will see that there is a problem with one category. Recall that we are doing RNA-seq analysis, not DNA sequencing. You should be curious about the problem you see. The way you get most information in the bioinformatics world is via the Internet using Google. Also, there are specialized bioinformatics help sites where you can post questions or read answer to already posted questions. Chances are VERY high that your question has been posed and answered. This is the case for your problem at hand. Try out one of these help sites and get familiar with it by searching on the site for an answer. (Hint: there is one). Start here: https://www.biostars.org/
20. Check some discussion entries but don't spend too much time on it.

**Step 2: Mapping the sequences to the reference genome using Bowtie and Tophat**

1. From here on you are back on the iPlant platform using Terminal. The next step in your RNA-seq analysis is to map the sequence reads to the genome, from which they originate (in this case tomato). After they have been aligned to the reference you can count how many reads were found in each sample and then ask if one sample had higher gene expression for a given gene compared to the other sample. In our case we have two replicates (bioreps) for each developmental condition (breaker (orange color) versus immature fruit). First we need to download the

reference genome from the tomato genome database and "index" it in such a way that the aligner software can find where chromosomes start and end, where genes are, and what genes are annotated with known functions etc.

2. We will get the genome data directly from the tomato genome database. Either go to the ftp website, copy the link location there or from here: ftp://ftp.solgenomics.net/tomato_genome/annotation/ITAG2.4_release/ITAG2.4_genomic.fasta
3. Then, **from the iPlant Desktop directory**, use wget to download the genomic sequence, which contains all genic, intergenic, and unsorted/unmapped regions of the genome. $ `wget ftp://ftp.solgenomics.net/tomato_genome/annotation/ITAG2.4_release/ITAG2.4_genomic.fasta`
4. Still in the Desktop directory, using the software Bowtie2, build the index using this command: `$ bowtie2-build -f ITAG2.4_genomic.fasta ITAG2.4_genomic_btindex` #Note that `bowtie2-build` is the command, `-f` an option specifying the input format as FASTA, the genome input file is provided in FASTA format, and at the end of the input the name of the output file is created, which in this case should be ITAG2.4_genomic_btindex. Make sure there is a space after build and before –f, and another space after .fasta and before ITAG2.4.
5. Depending on network traffic and connection speed, this will take about 20 minutes to process. (While waiting for Bowtie, skip to step 7 and write the tophatin script, see below.) The Bowtie software produces a number of files all ending in .bt2. Later on the next software will use a hidden wildcard to find all of these .bt2 files as it needs them without you having to specify each.
6. Once Bowtie finishes, check that the btindex files are in your Desktop directory. If they are not already there, move all btindex files onto your Desktop using `mv`.
7. Check that you are **in the Desktop directory** and make a new script called tophatin.sh (using `touch`, `chmod`, etc.).
8. Write the script for all four files (breaker *334, *336 and immature fruit *331, *333). The tophat2 command looks like this. **Note that there is a space after "breakerXXX" (or "immatureXXX"), and a space after "btindex".**

```
$tophat2 -o tophatout_breaker334 ITAG2.4_genomic_btindex
/home/johntester/Desktop/Bio332_BTI_exercise_data/SRR404334_ch4.fq
```

(Double check that you spelled the word *exercise* correctly in the script.)
For each file use a separate line of code separated by a return. The –o option specifies the name you want for the output file. Here we will use tophatout_breaker334 for the first file. The other files have to have the out option according to the input file name, in other words breaker336 or immature331 or 333, otherwise you will overwrite the file each time you reuse the same output file name. After the space put the prefix of the bowtie index files that you made in the previous steps. All you need is to write the prefix as you see in the example. Then specify the absolute path to your input files. The only thing you need to change is the username (if you named all the directories and files as suggested in this manual and spelled them correctly). Note that there is exactly one single space

between btindex and /home/… and nothing else (the line break is only for readability in this document). Each individual tophat command will take ~ 25 minutes to complete (x4 = ~2 hours total).

`Ls –l`

9. To run your script with all four lines of code use the screen command
   $ screen -L ./tophatin.sh #Note that you cannot run this script until Bowtie finishes and produces the files tophat needs.
10. You can now either wait 2 hours or turn off the computer and open it back up later at home.
11. Once Tophat finishes you should have four tophatout* directories, each of them with multiple files of alignments that you will later need.
12. Verify that this is the case before the next lab period (or you will have no files to work on next week) as follows: Use `cd` to move into each tophatout* directory and check that each of them has a file called "accepted_hits.bam".
13. Go back to the login webpage for iPlant Atmosphere. If you have used up more than 50% of your allotted AUs (see the bar on the left under "My Resource Usage"), click on "Request more Resources" and in the two boxes ask for "200 more AUs" "for a workshop". It will take up to a day (or over the weekend) to get the AUs.
14. Once you have verified that Tophat has finished and worked, make sure to ***suspend your instance*** on the iPlant website or you will run out of CPU hours.

# Bio332
# Bioinformatics lab: Introduction to command line computing with iPlant using the Tuxedo pipeline for RNA-seq (lab 3)

**Step 3: Assembling reads and quantifying expression levels with Cufflinks**

1. Login to Atmosphere, move to the Desktop directory and use ls to check for the tophatout* directories from the previous step.
2. Use cd to move into each tophatout* directory and check that each of them has a file called "accepted_hits.bam".
3. Then move back into the Desktop directory.
4. Next you need to write a script for inputting the accepted_hits.bam files into the Cufflinks software. Using `touch`, `chmod`, and the `emacs` text editor commands, create an executable script, called cufflinksin.sh, that contains four lines of code (two biological replicates for each of two conditions breaker and immature fruit). The command for the first line is composed of the following elements:
   ```
   $ cufflinks -o cufflinksout_breaker334
   tophatout_breaker334/accepted_hits.bam
   ```
   The command is cufflinks, the option –o tells cufflinks that you want to specify the directory name it will produce for the output (here you specify cufflinksout_breaker334 as your first directory). Following a space (not a line break), you tell cufflinks where to find the input file for the command. In this case it is the accepted_hits.bam file in the tophat_out334 directory. Note: each line of code in the script has to specify a different output directory name, or else the same directory will be overwritten every time the script moves to the next line of commands to execute. Also notice that the file "accepted_hits.bam" has the same name for each of the four different data sets (*334, *336, *331, and *333). Therefore it is very important that you specify the correct directory these files are located in. Also note that this will only work if you are in the Desktop directory – otherwise the path to the files is incomplete and you will get a "no such file or directory" error message.
5. If you need help writing the script take a look here. (Note that inside a script you don't have a $ sign at the beginning of a line)
   ```
   cufflinks -o cufflinksout_breaker334 tophatout_breaker334/accepted_hits.bam
   cufflinks -o cufflinksout_breaker336 tophatout_breaker336/accepted_hits.bam
   cufflinks -o cufflinksout_immature331 tophatout_immature331/accepted_hits.bam
   cufflinks -o cufflinksout_immature333 tophatout_immature333/accepted_hits.bam
   ```
6. When your script is ready, run it with `$ screen -L ./cufflinksin.sh` This complete dataset should take about 1 hour and 20 min to run on your iPlant instance using 1 CPU. (You can already do step 8 while you wait.)
7. Once done, look at each directory to check if it has the expected files inside:
   ```
   genes.fpkm_tracking  isoforms.fpkm_tracking  skipped.gtf  transcripts.gtf
   ```
8. Before you can merge the four assembly files that you got from cufflinks into one assembly that will have all of the expressed genes from all treatments against which you will later compare each individual sample, you need to write a small input file that the software "cuffmerge" needs in the next step. You also need to download an annotation file from the tomato genome database, called a .gff3 file. This file contains all gene model annotations known for all genes in the genome.

A gene model describes for example the number of exons, the intron/exon splice junctions etc.
First the small input file: using `touch`, `chmod`, and `emacs`, write a file with four lines that specifies the location of the transcript files and call it assemblies.txt. Make sure the assemblies.txt file is in the Desktop directory.

```
./cufflinksout_breaker334/transcripts.gtf
./cufflinksout_breaker336/transcripts.gtf
./cufflinksout_immature331/transcripts.gtf
./cufflinksout_immature333/transcripts.gtf
```

9. Next download the .gff3 annotation file from [ftp://ftp.solgenomics.net/tomato_genome/annotation/ITAG2.4_release/ITAG2.4_gene_models.gff3](ftp://ftp.solgenomics.net/tomato_genome/annotation/ITAG2.4_release/ITAG2.4_gene_models.gff3) into the Desktop directory using `wget`.
10. Now, use the following command to merge the four transcript files.
`$ cuffmerge -g ITAG2.4_gene_models.gff3 -s ITAG2.4_genomic.fasta assemblies.txt` # The –g option specifies the genome annotation file that follows (the .gff3 file you just downloaded). The –s option specifies which genomic DNA file to use as reference genome. Following a space, the assemblies.txt file has the location for the 4 individual transcript assemblies (see above). This operation will take about 40 minutes to complete. The output directory is one directory called "*merged_asm*" and contains the merged assemblies of all conditions and bioreps.
11. Check that you have a directory called *merged_asm* on the Desktop. Use cd to enter the directory and look if you have a file called merged.gtf. Move back out into the Desktop directory.

**Step 4: Identify differentially expressed genes between treatments/conditions using Cuffdiff**

1. The Cuffdiff command is best run in screen from a shell script. Using `touch`, `chmod`, and `emacs`, make an executable script called *cuffdiff_in.sh* in the Desktop directory.
2. The cuffdiff input is quite complex. Look at the command first:

```
$ cuffdiff -o diff_out -b ITAG2.4_genomic.fasta -L immature,breaker -u merged_asm/merged.gtf ./tophatout_immature331/accepted_hits.bam,./tophatout_immature333/accepted_hits.bam ./tophatout_breaker334/accepted_hits.bam,./tophatout_breaker336/accepted_hits.bam
```

The option –o specifies the output directory's name to be made (*diff_out*). Option –b specifies the reference genome. –L lists the names of the two conditions that you later want the graphics to use (here "immature" and "breaker"). Option –u specifies the merged alignment file. Then there is a space and a list of bioreps for each condition (we only have two bioreps). The ./ specifies that the path starts in the present directory. So cuffdiff will look for the *tophatout_breaker334* directory, open it and look in it for the file "accepted_hits.bam". The bioreps are separated by a comma (no space after the comma). The two conditions (strings of bioreps) are separated by a space (no comma). There is also a space after the –L and after the –u.

3. Place this command in the cuffdiff_in.sh script. (This needs to be typed into the script because of "hidden" characters in the Word document, such as line breaks, returns etc.)

4. Execute the script as follows: `$ screen -L ./cuffdiff_in.sh`
5. With only one CPU running, this program will take about 180 minutes to run. At the end you should have an output directory called "*diff_out*" in your Desktop directory. Open the directory and check that it has the following files:

```
[johntester@172 diff_out]$ ls
cds.count_tracking      genes.count_tracking    isoforms.read_group_tracking  tss_groups.count_tracking
cds.diff                genes.fpkm_tracking     promoters.diff                tss_groups.fpkm_tracking
cds_exp.diff            genes.read_group_tracking  read_groups.info  tss_groups.read_group_tracking
cds.fpkm_tracking       isoform_exp.diff        run.info          cds.read_group_tracking
isoforms.count_tracking   splicing.diff   gene_exp.diff   isoforms.fpkm_tracking   tss_group_exp.diff
```

6. Go back to the login webpage for iPlant Atmosphere. If you have used up more than 50% of your allotted AUs (the bar on the left under "My Resource Usage"), click on "Request more Resources" and in the two boxes ask for "200 more AUs" "for a workshop". It will take up to a day (or over the weekend) to get the AUs.
7. Once you have verified that Cuffdiff has finished and worked, make sure to ***suspend your instance*** on the iPlant website until you come back to do the last part or you will run out of CPU hours.

**Notes:**

If files need to be transferred between two iplant accounts, the following command can be adapted:

```
[johntester@vm65-168 Desktop]$ scp -r
johntester@128.196.65.168:/home/johntester/Desktop/tophatout_breaker336
/accepted_hits.bam
nlouisesmith@128.196.65.169:/home/nlouisesmith/Desktop/tophatout_breake
r336

Note the spaces after $, after scp, after -r, after …bam (and nowhere
else)

General usage is:
scp user@sourceservername:sourcefilepath
user@destservername:destdirectory

use option -r for entire directories
```

## Postlab

(One set of answers per individual, please. Due 2 days after lab by email.)

If you haven't read the reading for this lab on Moodle (Trapnell et al., 2012, Differential gene and transcript expression analysis of RNA-seq experiments with TopHat and Cufflinks Nature Protocols, 7, 562-578), get the paper now to answer the questions. To answer d) and e) you need to read ahead in the manual for next week.

For each of the following five major steps of the analysis pipeline, explain in 1-2 sentences what each step accomplishes in the grater scheme of the analysis. Do not copy verbatim from the paper but answer in your own words. It is important that I can tell from your answer if you understand the principal behind each individual step.

a) Bowtie
b) Tophat
c) Cufflinks package
d) CummeRbund
e) TopGO

# Bio 332
# Bioinformatics lab: Introduction to command line computing with iPlant using the Tuxedo pipeline for RNA-seq (lab 4)

**Note: Read the postlab assignment on the last page of this lab so you know which files to save.**

Log into Atmosphere and navigate to your desktop files.

**Step 5: Visualizing differential gene expression from CuffDiff output in R**

1. The visualization of the data files you produced using the Tuxedo pipeline happens on your own computer (not the iPlant cloud computer) using the software R. If you have R on your computer, check that the version is up to date (you want version 3.1.2 for Mac). Install the free software R on your computer as follows. Note that R-Studio may or may not be sufficient. Also note that you do need to have the newest version of R. If your version is old it may not be compatible with the packages you will later download. If in doubt, download the latest version.
   Go to [http://cran.fhcrc.org/](http://cran.fhcrc.org/)
   Click on the Download button for Mac or Windows (depending on the type of machine you use). Read the instructions and install the software on your hard drive. Once the file is downloaded to your download file (it will take 2-3 minutes, then look there for it), double click on the file, click "continue", "agree", and "install". Type in the password for your computer (not your iPlant password), so you can install the software.
   R uses a prompt that looks like this: >, whereas the Unix prompt is this: $
   Once R is installed you will need to install several more packages. Start with "Bioconductor": open R and type in after the > (don't type the >)
   `> source("http://bioconductor.org/biocLite.R")` . Hit return.
   When R finishes, type in `> biocLite()` and hit return. If R asks if you want to update something, respond with `a` (as in "all").
   Now install the software package cummeRbund by typing after the prompt:
   `> biocLite("cummeRbund")` # wait a few minutes even if the computer seems to be not responding.
   Type `> library(cummeRbund)` to see if the software is loaded.
2. First you need to download the gene expression differences file called *"gene_exp.diff"* that is inside the directory *diff_out* from your iPlant account to your own hard drive using a unix command called `scp`. Since you will need more files out of that directory later, we will copy the entire directory.
3. Open a new (second) terminal window on your machine (not on the virtual iPlant machine).
4. Modify the following command to fit your username and IP address.
   ```
   TH223F-6657:~ amadlung$
   scp -r johntester@128.196.64.198:/home/johntester/Desktop/diff_out .
   ```
5. NOTE: at the end after the diff_out there is a space and then a dot. This means you want the file to be copied with the same name to directory on your hard drive from which you issue the scp command, aka the "current directory". The option –r is needed when copying a whole directory, not just a file, as you are doing here.

6. On your computer, go to your home directory (in Macs use your mouse to go to Finder and then click on the house icon) and locate the folder you downloaded called "*diff_out*". Drag it onto your desktop.
7. If you haven't already, start R now and open the cummerbund library
`> library(cummeRbund)`
8. The library should load (if it isn't already loaded).
9. Next you have to create a database file that R will use later on repeatedly to draw the graphs you will want. Use this command:
`> cuff_data <- readCufflinks ('diff_out')` #**NOTE**: You will have to replace '*diff_out*' with the correct file path for your computer. If you followed each step as described above your file path should be:
`'/Users/yourusernameforyourcomputer/Desktop/diff_out'`
Make sure you replace yourusernameforyourcomputer with the username of your own computer, not the virtual iPlant username (if you don't know your username open a terminal window and look before the $. You can also help yourself in R by using tab-complete. R will give you the choices it finds on your computer). Also, take note that there is an underscore in the diff_out folder. On my computer the command looks like this:
`> cuff_data <- readCufflinks ('/Users/amadlung/Desktop/diff_out')`
10. R will create the database. This will take a few minutes.
11. Now you can use the database to draw a variety of figures and write tables to illustrate the data.
12. Type > `cuff_data` for a list of descriptive statistics about your data set.
13. We are of course interested in those genes that are statistically significantly differently expressed between immature fruit and fruit at breaker stage. Use the following series of commands. Note: R uses `<-` to assign a series of data to a new file name. The new file name is left of the `<-`. Note also how the new file name is then used in the second line again to provide the subset of data that go into the next file of only the significant genes.
`> gene_diff_data <- diffData(genes(cuff_data))` #This makes a file with gene expression differences.
`> sig_gene_data <- subset(gene_diff_data, (significant=='yes'))`
# This makes a file with gene expression differences of only those genes that are also passing a statistical test of significance. Notice that there are two = signs after "significant".
`> nrow(sig_gene_data)` # now list the number of significant genes in the sample.
**Question to answer:** How many significant genes are in your data set? ________

14. Next we will graph the data, still using R. After you type the command below and hit return, a new window will open with the scatter plot. Genes with differential expression are those not on the line.
`> csScatter(genes(cuff_data), 'immature', 'breaker') +`
`geom_point(aes(scale_size=4, scale_shape=19))`
#Note that after the + sign you need to hit RETURN (the prompt on the next line will change from a > to a +, which means that R expects more information to follow. Or provide the command on one line.

15. A new window will open (the Quartz viewer) displaying the expression values of each gene for the two conditions. Any gene not differentially expressed between the two should fall along the line. Any different genes will be above or below the diagonal line.
16. Now that we have a visual impression of the results, let's get a list of all the genes that are significantly different, so we can start to understand what types of genes are affected.
```
> write.table(sig_gene_data, 'diff_genes.txt', sep='\t',row.names
= T, col.names = T, quote = F)
```
17. Go to your home directory where the gene list is deposited with the name "*diff_genes.txt*". You can open it as a text file, but it is easier to see as an Excel file. Now that the list is short, we can use Excel again. Go to Excel on your computer, go to "open" and then navigate to this file and open it in Excel. Excel will ask you how you want to import the .txt file. Click on "next" twice, then "finish" and Excel will put your tab-delimited text file into a regular Excel spreadsheet. We won't need this exact list right now, but later you will need to know how to download a similar list and expand it into an Excel file. For right now, look at the column headers and see if you can find the last column that tells you if a gene is significantly differently expressed between immature fruit and fruit at the breaker stage (should say "yes" or "no").
18. There is a problem with the Excel list! On the left hand side you see two columns of gene names and identifiers. Unfortunately, those are not the gene identifiers (GeneIDs) that are currently being used in the tomato database anymore. Go to your Terminal window and open the original file from which this list of differentially expressed genes was extracted (Desktop/diff_out/gene_exp.diff) with `less`. You will see that there is a third column with gene names in the form of Solyc04gxxxx. That is the gene identifier we need (Solyc stands for *Solanum lycopersicum*, 04 for chromosome 4 and g for genomic content, not chloroplast or mitochondrial). How can we extract the data we want (all those that are significant) from this file without having to look at it by hand? The instructions for cummeRbund do not have a command that extracts the columns that we want. We can use Unix to extract the lines or columns we need. The `grep` command allows us to extract rows (always entire rows) that contain something all rows we want have in common. For example, you could extract all rows with gene names starting with or containing the word "Chromosome 1" (if there were any in our example).
19. Open the file again in Terminal with less and look if there is something that might be useful to us in extracting all those rows that are significant (compared to those that are not).
20. You might have noticed the column that lists "no" (or "yes") for significance. That is the handle we can use to filter the rows we want. `grep` has a specific pattern of usage:
```
$ grep ‘stringtolookfor’ filetouse > newnameoffiletomake
```
21. Make sure to use the single quotes for the string and use spaces but no commas. Here is a command you can use:
```
$ grep 'yes' gene_exp.diff  > grep_yes_gene_exp.txt
```

22. Using the command `wc`, determine how many significant genes (=lines in the file) there are. (The three values provided by `wc` are lines, words, and bytes)
    **ANSWER**: There are ____________significant genes (lines of text)
23. The new file should be on your computer's hard drive on the Desktop in the diff_out directory. Open it up with Excel as before. Now you have the gene list of differentially expressed genes with gene identifiers that we can use in the next step.

**Step 6: Graphing gene expression for specific genes from the data set using R**

1. Let's get a bar graph for the expression of single genes that we might be specifically interested in in our data set. We will use the first gene on the list: XLOC_011676
2. Use the following set of commands to first define the gene, tell R to get the information for that gene from the database you made earlier and then graph it in the Quartz viewer that you have used before and that automatically opens.
   > XLOC_011676 <- getGene(cuff_data, 'XLOC_011676') # "getGene is the command, XLOC_011676 is the file name for the info for that gene, left of <-, and the same name is used in ' ' within the ( ) to tell the command what row to look for in document cuff_data.
   > expressionBarplot(XLOC_011676) # Now the command is "expressionBarplot" and the argument from which to make the bar plot is simply the file name we specified in the previous command. Hit return and look for the quartz viewer window on your desktop. This command even gives you the Solyc gene name on the bar plot.
   Usually you will want to show more than just one gene. You can display the list of significant genes in R. You already made a file earlier that you called sig_gene_data (Step 16 on previous section). Just for fun, stretch your R window wide and type > sig_gene_data and hit return. You should see the entire file displayed. (Do not do this if your list is thousands of lines long or you will have a long list to scroll through.)
3. In the next step we will use the list of significant genes as input to graph or display all significant results in a heatmap. For that you will first need to create a text file that only contains the list of gene_id names. Your *grep_yes_gene_exp.txt* file (in your diff_out directory on the Desktop) contains one column called gene_id. We will need to get this column into a text file without any formatting. Theoretically we could open the diff_genes.txt file in Excel, copy and paste the column into a new text file and use that. Sometimes that might work and sometimes it may not, because Excel uses underlying invisible characters (like tab formatting, line end returns etc.) that will give us problems later. It is easier to do this with a few simple Unix commands. First, in your terminal window for your own (non-virtual) machine, create a file (using `touch`) inside your diff_out folder called *sig_genes_grep_yes.txt*.
4. Now use the `cut` command (see your unix cheat sheet) as follows:
   th223f-6657:diff_out amadlung$ `cut -f 2 grep_yes_gene_exp.txt > sig_genes_grep_yes.txt` #Note that you need to be inside your diff_out folder, like in this example or it won't work.

5. Check the file you made using `less`. You should have a list of XLOC identifiers.
6. Now that you have the file with the gene identifiers of significantly differentially regulated genes you can go back to R and use the cufflinks data to create more analysis graphs and figures. First, R needs to extract the subset of genes you want to display (in this case the significant ones) from the data set R has already made in steps 5.12 and 5.13. Using the R console, type the following command. Make sure to use your own username instead of “amadlung”. If you get an error “no such file or directory”, check that your file is in fact in a directory on your Desktop called diff_out.
```
> mySIGGENEIDS <-
read.table("/Users/amadlung/Desktop/diff_out/sig_genes_grep_yes.txt")
```
# the new file you are making in R is the one called mySIGGENEIDS; read.table is the command to R to read the text file with your input gene IDs (called sig_genes_grep_yes.txt).
7. Check that the file was made correctly by displaying it:
`> mySIGGENEIDS` # You should have 83 genes.
8. Next, use the `getGenes` command in R to create a smaller database that has the expression data (and a lot more information) only for your significant genes.
```
> MYSIGGENES <-getGenes(cuff_data,mySIGGENEIDS[ ,1])
```
# This file uses the file mySIGGENEIDS (with just the list of significant genes) and pulls data from the database made in steps 5.12/5.13. The command is getGenes. The [ ,1] portion is needed to specify which rows and columns of that file you want. Leaving the space before the comma blank means “all rows”. The “1” after the comma means: “use column 1”.
9. R will now spit out ~25 rows of red text before returning the > cursor.
10. Type > `MYSIGGENES`
11. R will provide some information on the data set you specified and extracted for the large database.
12. Make a heatmap of the genes that are significant using these commands:
```
> h <-csHeatmap(MYSIGGENES, clustering="row", heatscale=
c(low='red', high='green'))
```
# Here the command is csHeatmap, the argument to be worked on is MYSIGGENES. The modifying addition heatscale = tells R something about your specific wishes for the optics of the heatmap. Ignore the red text R returns with information and type
> `h`
14. If the resulting map is a bit cluttered you can make the font of the names smaller by adding a definition to the plot instructions:
```
hm <- h +
theme(axis.text.x=element_text(size=14),axis.text.y=element_text(
size=6))
```
#Hit return and then issue the following command:
> `hm`
15. R can now also use the list of genes specified above for a barplot of all significant genes simply using `> expressionBarplot(MYSIGGENES)`
16. To customize the barplot use the following commands:
```
> b <-expressionBarplot(MYSIGGENES)
> bm <- b + theme(axis.text.x=element_text(size=6),axis.text.y=element_text(size=10))
> bm
```

17. Now let's create a new subset of genes we want to display. We will use the 10 genes starting at XLOC_012549. First, create a new subset .txt file from the file sig_genes_grep_yes.txt. Create a file using `touch` called sig_genes_grep_yes_10.txt

18. Now use `grep` as you did before to extract only 10 lines of text. We will use the option –A. The number following –A is the number of lines you want displayed after `grep` detects the first word (called a string) you specify. Try this command:

```
$ grep -A 10 -i "XLOC_012549" sig_genes_grep_yes.txt
```

Your terminal will display the 10 lines starting with XLOC_012549. To direct them into the new file you just made use this command instead:

```
$ grep -A 10 -i "XLOC_012549" sig_genes_grep_yes.txt >
sig_genes_grep_yes_10.txt
```

19. Use the following command to create a new R object in the R console containing the 10 genes you want to display:

```
my10SIGGENEIDS <-
read.table("/Users/amadlung/Desktop/diff_out/sig_genes_grep_yes_10.txt")
```

20. Verify using > my10SIGGENEIDS

21. Now use the getGenes command to populate the file with the data form the database:

```
> MY10SIGGENES <- getGenes(cuff_data,my10SIGGENEIDS[ ,1])
```

22. Verify using `> MY10SIGGENES`

23. Create a new object to display later:

```
> b10 <-expressionBarplot(MY10SIGGENES)
```

24. Specify the fonts etc.

```
> bm <- b10 +
theme(axis.text.x=element_text(size=6),axis.text.y=element_text(size=10))
```

25. Display the graph using `> bm`

26. **ANSWER THIS QUESTION:** One gene appears to be completely off in immature fruit and transcribed in early ripening fruit. Which gene is it?

**Step 7: Determining functional categories for the significant genes**

To determine the general functions of a selection of genes, often a gene ontology, or GO search is performed. There are multiple tools that will do GO analysis and each has their pros and cons. We will use an R package called TopGO.

GO analyses can accomplish multiple things: usually the first thing you want to do with a list of significant genes from an experiment is to see what functional categories your significantly differentially expressed genes fall into. For most sequenced genes there is an annotated list available that contains the gene IDs, the gene names, their functions, and also their GO category. You can then compare your list of significant genes with the GO term list, pull out the GO terms for your genes, and do a statistical analysis that determines if the number of genes per category on your list of significant genes is particularly high in a given functional category, and thus suggests that these categories are particularly affected by the treatment in your experiment. In that case you can say that GO category X is "overrepresented" among your significant genes. The test you run for that is called a Fisher's Exact Test. Let's briefly look at an example of GO overrepresentation: Let's assume your genome has 10,000 genes and your treatment found a total of 1000 genes to be differentially regulated using RNAseq analysis. 1000

out of 10,000 is 10%. Now let's assume a particular GO category (say glucose metabolism) contains a total of 100 genes. Given that you found 10% of genes to change in your experiment you could argue that every category might be equally affected and thus you might expect 10% of each category to be differentially expressed. For the example category (glucose metabolism) you would expect 10 genes to be significantly different (100 genes in category, 10% rate of overall change = 10 genes in this category). Let's say a different category has only 30 genes in it, so for that the expected change would be 3 genes (also 10%). Let's now say your experiment found that among the glucose metabolism genes not 10 but 50 genes were affected, then that is a lot more than you would expect if all categories were equally affected (that's your null hypothesis). 50% instead of 10% of genes affected from the glucose metabolism GO category suggests that clearly this GO category is overrepresented among the categories containing your significant genes. Maybe something is going on with this entire pathway that you might now want to investigate more carefully in a follow up experiment. You could now form a new hypothesis based on your GO analysis using your RNAseq data. But what if your level of change in this category was 15 genes (As opposed to the expected 10 among the total of 100) – is that a significant overrepresentation? When it isn't as clear as in the previous example, you need to employ statistical tests, and that is where we will use the Fisher Exact test.

What you will do in this analysis is two things:

a) Determine the GO categories for your significant genes
b) Determine if there are GO categories that are statistically significantly overrepresented among the GO categories of your genes (that were to be found significantly differentially regulated) using Fisher's Exact test.

1. We need two files: the file that contains all genes in the genome and their GO categories, and the file that contains a list of the gene names that were interesting (significantly differentially regulated) in your RNAseq analysis. Let's get the first file first. Go to the tomato genome database and download a file that contains what you need: gene IDs and GO categories. The file is on their ftp site. Download it to your physical (not iPlant) desktop using the curl command. The URL is:
ftp://ftp.solgenomics.net/genomes/Solanum_lycopersicum/id_conversion/tomato_unigenes_solyc_conversion_annotated.txt
As you download the file, name it tomato_goterms.txt

```
$ curl ftp://ftp.solgenomics.net/genomes/Solanum_lycopersicum/id_conversion/tomato_unigenes_solyc_conversion_annotated.txt >
tomato_goterms.txt
```

2. Open the file using Excel and look at the many different columns. You will need columns 2 and 15. We will need to cut the file to size using unix commands. Close the excel file.

3. Go to your terminal window and check if you have the file on your physical computer desktop. Then cut columns 2 and 15 and place them into a new file called tomato_goterms_only

```
$ cut -f 2,15 tomato_goterms.txt >tomato_goterms_only.txt
```

4. Now open this file up in Excel. Next we need to delete the last two digits of the Solyc gene ID to have the same gene ID format as in the list from the RNAseq data. To do so,
a) insert a new column (Insert → columns) left of your geneID column, then
b) click on the top cell in the empty new column (column A, row1) and type the following function:

=LEFT(B1,16)

This function returns the string of letters and numbers from cell B1 up to the $16^{th}$ digit and places it in cell A1 where your cursor is.
c) Click and drag the cursor from cell A1 to the bottom of column B so that all cells in column A are selected up to the bottom of column B (while keeping the cursor in column A), let go and go to Edit → Fill down. (You can do this also by double clicking on the lower right hand corner of the A1 cell after typing in the formula. It will fill down to the end of the data in the adjacent column.)
d) Now highlight columns A-C with the mouse, and click Data → Sort → Sort by col. C.
e) Now scroll down to where the GO numbers end and delete the geneID rows without GO number. that's quite a few that don't have functional annotation yet (about 12K).
f) Now save the file as *tomato_goterms_only_edit.txt* BUT MAKE SURE to set the "Format" pull down tab in the save window to "Windows Formatted Text (.txt)" or your file will not work and later give you a lot of gibberish with inserted ^M characters in your file.
g) Now you must delete the column with the old Solyc numbers. That might require you to copy and "paste special" the values of the new column in yet another column, before you can throw away the old columns, leaving you with exactly 2 columns: The new Solyc gene IDs and the GO categories.

5. Go to your terminal window and open the file you made and edited:

```
$ less tomato_goterms_only_edit.txt
```

Check that it is clean with no extraneous characters. If that is the case, this concludes formatting your first input file!

6. Now we need to create the second input file, which needs to contain all significant genes listed by their gene ID (Solyc.xxxxx number). Using grep we already made a file earlier that contains all significant genes that we called "grep_yes_gene_exp.txt". It should be in your diff_out folder. Check that you have this file and open it.

7. Next we will cut the column we want, and place it in a new file that we will call sig_genes_Solyc.txt as follows:

```
$ cut -f 3 grep_yes_gene_exp.txt > sig_genes_Solyc_clean.txt
```

8. Using Excel, open the file you just made. Click "next" twice and then "finish". The list of significant genes (Solyc numbers) needs a bit of tidying up, which we will do by hand. Each row should have one number only. You can achieve this by cut and paste. Either add the additional numbers to the bottom of your list or create new rows (→insert → row). When you are done, double check that you have deleted the extra commas in rows

that had several gene names as well or the file won't read in properly in the next step. You should have to make about ~7 changes and end up with a list of about 86 genes. Save the file as "windows formatted text (.txt)" in the pull down menu. (Other formats insert hidden characters and mess up your file for the next step).

9. Back in Terminal, go to the diff_out folder on your physical desktop and, using ls, look for the file

sig_genes_Solyc_clean.txt

and open it with less. You should see one column of Solyc gene IDs with a suffix ending in .1 or .2 (just like your Solyc number in your input file 1 from steps 1-5 above).

10.Next we will use R to do the following:
a) create tables sorting the significant genes of the RNAseq analysis into GO categories,
b) conduct two types of Fisher Exact tests, one "classical", and one that weighs adjacent GO categories differently from those that are far apart in the GO hierarchy functional network.
Open R Studio on your computer now. Open a new R-script (File → New → R-script)

11. Copy and paste the script below into the empty top left pane.

```
##START COPYING HERE
### Script written by Keisha Carlson, edited by Andreas Madlung
#July 9th 2015
#make sure to have biocoductor, and topGO packages, need to load separately Rgraphviz
#for graph only
source("http://bioconductor.org/biocLite.R")
biocLite("topGO")
library(topGO)
biocLite("Rgraphviz")

###This set of commands analyzes the list of significant genes and tests if they are
#overrepresented in their
##respective categories of gene functions.

#read in the text file with column 1 = solyc numbers and column 2 = GO IDs into topGO
#format
gene_GO_map <- readMappings("/Users/amadlung/Desktop/tomato_goterms_only_edit.txt")
#make a list of solyc numbers
geneNames <- names(gene_GO_map)
#open up file with genes of interest: file contains 1 column of solyc numbers of
#differentially expressed genes from RNAseq analysis
myINTgenes_file <- read.table("/Users/amadlung/Desktop/diff_out/sig_genes_Solyc_clean.txt", header = T)
#take column 1 (only column, but now no longer a table)
myInterestingGenes <- myINTgenes_file[[1]]
#takes gene names and adds 1 or 0 depending whether "interesting gene" or not
geneList <- factor(as.integer(geneNames %in% myInterestingGenes))
names(geneList) <- geneNames
```

```
#make topGO data object GOdata using biological process gene ontology and own
#annotations (from solgenomics.org)
GOdata <- new("topGOdata", ontology = "BP", allGenes = geneList, annot =
annFUN.gene2GO, gene2GO = gene_GO_map)
#run Fisher's exact test using "weight" algorithm
resultFisherWeight <- runTest(GOdata, algorithm = "weight", statistic = "fisher")
#run Fisher's exact test using "weight01" algorithm
resultFisherDefault <- runTest(GOdata, statistic = 'fisher')
#run Fisher's exact test using "classic" algorithm
resultFisherClassic <- runTest (GOdata, algorithm = "classic", statistic = 'fisher')
#get classic table, specify how many GO categories you want in "topNodes" (here e.g. =
#20)
classicTable <- GenTable(GOdata, classicFisher = resultFisherClassic, orderBy =
"classicFisher", topNodes = 20)
#get weight table
weightTable <- GenTable(GOdata, weightFisher = resultFisherWeight, topNodes = 20)
#get default table
defaultTable <- GenTable(GOdata, weight01Fisher = resultFisherDefault)
#make graph with network, specify number of categories you want using firstSigNodes
showSigOfNodes(GOdata, score(resultFisherWeight), firstSigNodes = 4, useInfo = 'all')
#Display results of classic table with topNodes and significance
classicTable
weightTable
#NOTE: Before the next step you should make two blank files on your non-virtual
#Desktop first and call them "Fisher_classic.txt", and "Fisher_weighted.txt".
#Next, write the table with the classic Fisher Exact test results to the file.
write.table(classicTable,"/Users/amadlung/Desktop/Fisher_classic.txt", sep="\t")
##Now write the table with the weighted Fisher results to the other file.
write.table(weightTable,"/Users/amadlung/Desktop/Fisher_weighted.txt", sep="\t")

##COPY ALL THE WAY DOWN TO HERE
```

12. Now read the script. You will have to edit the input path by taking out the path that works on my computer and change it to the path that will work on yours.

13. Now you can run this script line by line. Read the comments starting with # (in green) that explain each step for you. In the bottom left pane monitor the progress of the script. If you get red text with errors you have to go back and troubleshoot. Most likely errors will occur if the files are not in the correct place.

14.Once you get to around line 38 (showSigOfNodes…) (line numbers may have moved depending on how you imported the script), look for a graph to appear in the bottom right panel. This graph displays the network of categories that are overrepresented in your dataset.

15. Carefully read the lines that instruct you to make two blank file on your Desktop (starting with "NOTE:…"), into which to import your two tables with the "classic" and the "weighted" GO categories.

16. After finishing the script, open each of the two files using Excel, move the header over one column (it imports incorrectly one column over) and look at the far right column, which contains the p-value of the classic or weighted Fisher exact test. How many GO categories are significantly ($p<0.05$) enriched in your dataset?

17. Go back to script line ~38 (showSigOfNodes…) and change the number of categories displayed from 4 to 2. Re-run the line (you may have to clean out the bottom right panel with the broom function first). Then re-set the value to 6 and see what changes when you again re-run the line.

## Postlab assignment (10 points)

(One set of answers per individual, please. Due: In lab one week after this lab.)

Produce three publication-quality figures and a table that could be used in the Results section of a paper you might write up about the experiment. Make sure the figures have adequate legends. Ask for help if you are unclear what should go in the legend.
**Figure 1: Heatmap** of all significant genes
**Figure 2**: **Expression bar plot** of significant genes (your choice if you want to show one gene or all genes).
**Figure 3**: Analysis of functional groups the significant genes fall into. Create a bar graph in Excel that shows the expected and observed GO categories of your data set (either one or the other Fisher analysis) using paired bars of only the statistically significantly enriched GO categories in your graph. If no categories are significant, present the top 5 categories with the lowest p-values.
**Table 1:** Create a table and description (remember, table legends go on top of the table, figure legends go below) of one of the two Fisher analyses (your choice). Make the table as long as it makes sense from the data you present. Point out the significantly overrepresented categories.
