# Supplementary material for: Assessing an effective undergraduate module teaching applied bioinformatics to biology students
Source: PLoS Comput Biol. 2018 Jan 11;14(1):e1005872. doi: 10.1371/journal.pcbi.1005872 (PMC5764237; doi:10.1371/journal.pcbi.1005872)
Supplement: S2 Supporting information — Questions 1–31 were asked of both students and experts. Questions 32–41 were only asked of students after completing the module. Questions for which experts achieved “consensus” (more than 66.7% of answers were answered either as 1 or 2, or as 4 or 5) are italicized. (PDF) [file pcbi.1005872.s002.pdf]

S2 Supplemental Material

Questions on student/expert questionnaire. Questions 1-31 were asked of both students and experts. Questions 32-41 were only asked of students after completing the module. Questions where experts achieved "consensus" (more than 66.7% of answers were answered either as 1 or 2, or as 4 or 5) are italicized.

Student and expert questions:

1. My curiosity about the living world led me to study at least some molecular biology.

2. I think about and make connections between the molecular biology I learn in class with situations in everyday life.

3. After I study an aspect of biology that requires computational approaches to solve and feel that I understand it, I have difficulty applying that information to answer questions on similar aspects of biology.

4. Knowledge in computationally intensive aspects of biology consists of many disconnected topics.

5. When I am answering a question that contains aspects of bioinformatics or computationally intensive biology, I find it difficult to put what I know into my own words.

6. To understand bioinformatics, I sometimes think about my personal experiences and relate them to the topic being analyzed.

7. If I get stuck on answering a question in biology that involves a computational approach on my first try, I usually try to figure out a way that doesn't involve much computation.

8. I want to study biology because I want to make a contribution to society.

9. If I want to apply a method or idea used for understanding one bioinformatics problem to another problem, the problems must involve very similar situations.

10. I enjoy figuring out answers to biology questions that require bioinformatics.

11. To learn bioinformatics, I mostly need to follow software commands and procedures.

12. Reasoning skills used to understand bioinformatics can be helpful to my everyday life.

13. It is a valuable use of my time to study some fundamental concepts in computer science that I may apply to computational analysis of biological data in the future.

14. If I had plenty of time, I would take a computer science class outside of my major requirements just for fun.

15. If I had plenty of time, I would take a bioinformatics class outside of my major requirements just for fun.

16. The subject of bioinformatics has little relation to what I experience in the real world.1

17. There are times I think about or solve a biology question in more than one way to help my understanding.

18. If I get stuck on a biology question that involves the use of bioinformatics or computational biology, there is no chance I'll figure it out on my own.

19. There is usually only one correct approach to solving a problem involving bioinformatics and large genomic data sets.

20. When I am not pressed for time, I will employ additional analytical/mathematical/statistical/computational methods on a biology problem until I feel like I have the best answer as to why something works the way it does.

21. Learning computational approaches that are not directly relevant or applicable to human health is not something I would prefer to invest much time in.

22. Mathematical skills are important for understanding biology.

23. Computer science skills are important for understanding biology.

24. I enjoy explaining biological ideas or concepts to others whose study requires intensive computational analysis (using bioinformatics or statistics).

25. This statement is used to discard the survey of people who are not reading the questions. Please select agree (not "strongly agree") for this question to preserve all your answers.

26. The general public misunderstands many biological ideas that require statistics, bioinformatics, or computational biology.

27. Nearly everyone is capable of understanding bioinformatics if they work at it.

28. I do not spend more than a few minutes stuck on a biology question that requires a lot of computation, bioinformatics or statistics before giving up or seeking help from someone else.

29. Biological principles are just to be memorized.

30. I study bioinformatics/biocomputing to learn knowledge that will be useful in my life outside of school.

31. For me, knowledge of how scientists use bioinformatics is primarily important so I understand facts that are based on such analyses as opposed to using bioinformatics myself to investigate the unknown.

Student-only questions:

32. My curiosity about learning more bioinformatics has increased as a result of the module used in class.

33. The module provided new insights to me about the uses of computer sciences in biology.

34. I found the computer work during this module downright enjoyable.

35. Only answer if you don't particularly enjoy computational analyses (otherwise leave blank): Although I don't particularly enjoy computational analyses I think that this module gave me a good idea of what scientists do with genome-size data sets.

36. As a result of this module I am now considering taking a (or another) computer science course in the future.

37. I much prefer wet-lab or field-work experimentation to projects that require lots of computational analysis.

38. I don't mind using the computer for statistical analyses but I don't enjoy Unix-based computation.

39. I like bioinformatics when using tools with user-friendly web-interfaces but do not like command-line style analysis.

40. I might not like command-line style analysis of large data sets but I am glad I got to learn some of it.

41. Basic knowledge of command-line style bioinformatics/computational biology is likely important for my future career (whether I like it or not).
