# Supplementary material for: Assessing an effective undergraduate module teaching applied bioinformatics to biology students
Source: PLoS Comput Biol. 2018 Jan 11;14(1):e1005872. doi: 10.1371/journal.pcbi.1005872 (PMC5764237; doi:10.1371/journal.pcbi.1005872)
Supplement: S3 Supporting information — These responses were recorded from students during a focus group analysis one week after the conclusion of the module. Questions were asked in seven separately conducted sessions using a standardized script. (PDF) [file pcbi.1005872.s003.pdf]

S3 Supplemental Material
Student free text responses about their experience with the module. These responses were recorded from students during a focus group analysis one weeks after the conclusion of the module. Questions were asked in 7 separately conduced sessions using the following standardized script:

Engagement questions:
1. What is your favorite topic/subject in biology?
2. What do you mostly use the computer for?

Exploration questions:
3. How much and what kind of experience do you have with Biocomputing (statistics/bioinformatics)?
4. How do you feel about quantitative/computational approaches to biology in general?
5. When you are faced with a research/homework problem that could be approached better computationally, are excited or willing to try out a new software or computational approach? If you don't, why not?
6. How do you feel about computer science, programming and command line computing?
7. How do you feel about the instruction of bioinformatics and biostatistics in the current curriculum?

Exit question:
8. Is there anything else you would like to say about whether or not bioinformatics should get more coverage in undergraduate biology instruction?

Some sample answers from students are provided here. Only minor edits were made for clarity and sentence structure.
Answers to question 6:
- "I'm planning on taking Intro to Computer Science this summer because after this lab I've seen how far behind I am. I felt going through this lab the second time I was actually starting to understand the ways in which the command line works - I wasn't just copying from the lab handout. I was understanding why these commands were occurring where. I found that interesting and exciting, so I want to push myself farther to gain experience with these systems."
- "If I was here another year, I would definitely take Intro to CS, but I'm not."
- "I have a lot of interest in the applications, but my interest ends there. CS in and of itself doesn't interest me."
- "I think CS is really cool so I'm definitely going to take a couple CS classes."
- "I think it will only have increasing utility in the future of bio & science & environmental science so I am working to embrace it. But having no familiarity with it and a general disdain for math in general makes that a source of tension for my learning."
- "I really like the power that Unix allows you to chop things up and do these things that would be really hard in Excel. "
- "When I found out that we had a bioinformatics lab that would involve command line I was terrified because I had never interacted with my computer on that level before and I'm

usually really bad with computers. And then getting into it, it was just so much more methodical and manageable that I had anticipated, and now I'm thinking of taking an intro to computer science class next semester so I can keep developing this different part of my brain that I've never tried to interact with, so I think that's pretty cool."

- "I think it's a skill that I'd like to learn more, but I don't practice it enough to retain my abilities and so I get frustrated because I forget the commands really easily because I don't do it often enough."
- "I really liked it and I'd really like to continue with it in the future. As far as applications in the future I think a lot of people are moving toward it especially when analyzing really big sets of data."
- "I usually hate when we have to use any of the programs in lab, but I actually decided I liked this when we got to the end of it. It was definitely a learning curve."
- "I still find it difficult to figure out what exactly we were doing besides just typing in things."

Answers to question 7:

- "The university owes it to their students to keep them competitive and work with their interests, especially how competitive it is right now, not just getting into grad school, but after grad program and getting a dream tenured position."
- "It seems like it wouldn't be all that unreasonable to include a little module somewhere in every one of the bio classes that has some command line interface starting in freshman year."
- "I would have liked to see more of [this type of analysis] in my college career. I felt like it was like: 'Hey look, you should do this'.
- "After learning about Tophat and Go and the Cufflinks and all of that, that applies to like one very specific thing that you might need. So how many other programs are there for other very specific things that someone might need. That opened my mind that people could be doing so much stuff with large data sets, and I got overwhelmed just trying to think of it. Having a better understanding of where certain things could be applied would be cool."
- "Bioinformatics would be great to have to broaden our base as far as where a lot of biology is going."
- "I wish [bioinformatics] was integrated more into the classes we are already taking because we have a lot of requirements. Had there been another class like Comp Sci for Bio, I wouldn't have been able to take it even if I wanted to. So if every class I've taken along the way had a lab or two that applied a [bioinformatics] concept and they built on each other, I'd be stoked."
