# Supplementary material for: Assessing an effective undergraduate module teaching applied bioinformatics to biology students
Source: PLoS Comput Biol. 2018 Jan 11;14(1):e1005872. doi: 10.1371/journal.pcbi.1005872 (PMC5764237; doi:10.1371/journal.pcbi.1005872)
Supplement: S1 Fig — N (students) = 51; N (experts) = 50. Unless reported in Fig 3 (which shows a subset of these data), student responses compared with those of the experts did not differ between pre- and postmodule testing. This figure shows responses to all questions, including those for which experts did not reach consensus (see Methods and Results). SPE, student response pre module; SPO, student response post module; XPT, expert response. (PDF) [file pcbi.1005872.s004.pdf]

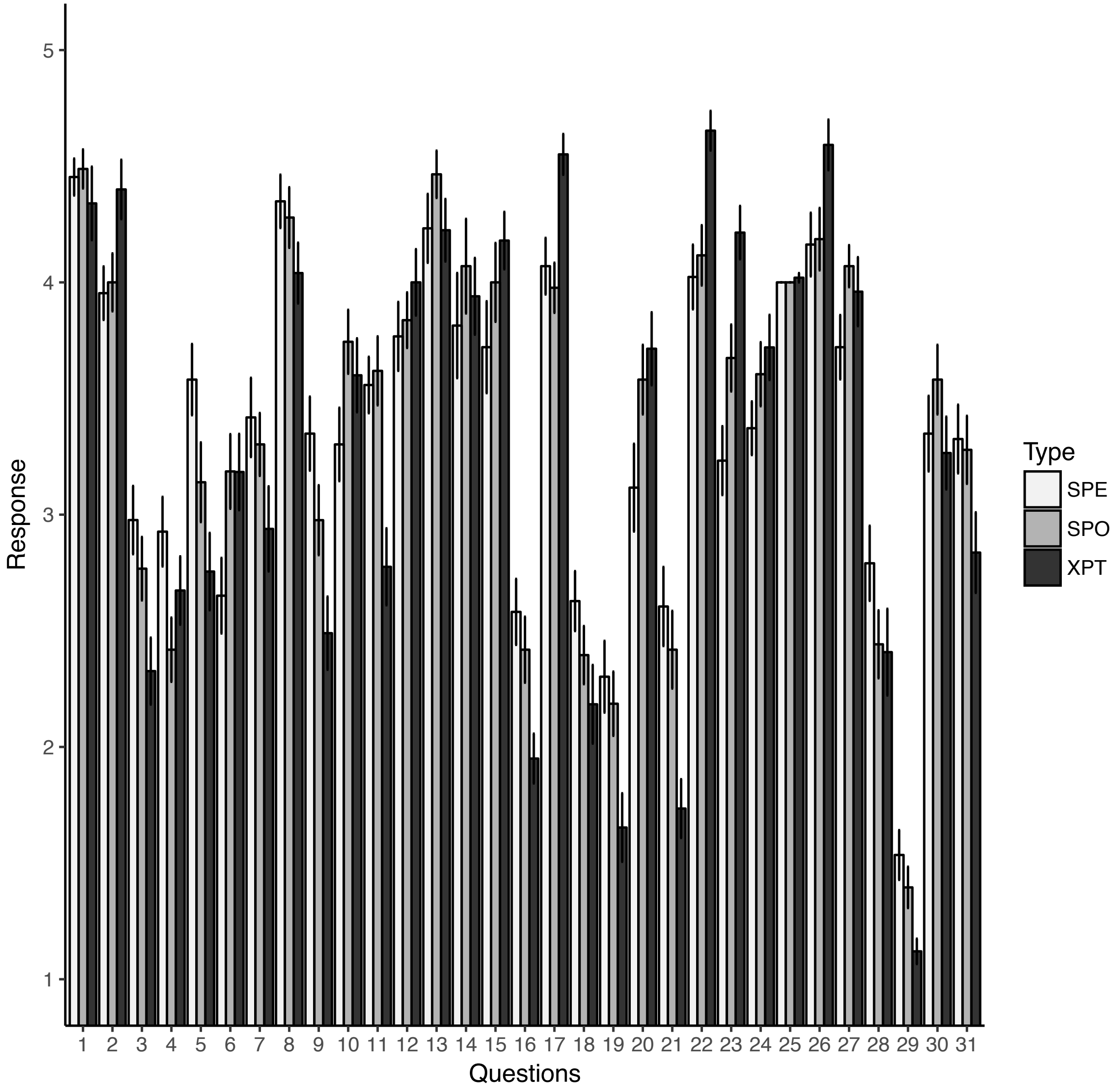

S4 Supplemental Fig: Student and expert responses to all questions on the questionnaire. SPE = student response pre-module, SPO = student response post module, XPT = expert response. N (students) = 51, N (experts) = 50. Unless reported in Figure 3 (which shows a subset of these data) student responses compared to those of the experts did not differ between pre- and post-module testing. This figure shows responses to all questions, including those where experts did not reach consensus (see Methods and Results).
